# Global Inventory of Fluoropolymer Production Plants and Their Associated PFAS Environmental Contamination

**DOI:** 10.1021/acs.est.5c18001

**Published:** 2026-04-08

**Authors:** Anna J. Miller, Kevin Kleemann, Juliane Glüge, Rainer Lohmann, Ian T. Cousins, Dorte Herzke, Mark F. Miller, Amanda Rensmo, Xenia Trier, Zhanyun Wang, Martin Scheringer

**Affiliations:** † 27219 Institute for Biogeochemistry and Pollutant Dynamics, ETH Zurich, Zurich 8092, Switzerland; § Graduate School of Oceanography, 54083 University of Rhode Island, Narragansett, Rhode Island 02882, United States; ∥ Department of Environmental Science, 7675 Stockholm University, Stockholm 106 91, Sweden; ⊥ NILU, Kjeller 2000, Norway; # Norway and Norwegian Institute of Public Health, Oslo 0456, Norway; ∇ National Center for Advancing Translational Sciences and U.S. Public Health Service, Research Triangle Park, Durham, North Carolina 27709, United States; ○ 4321 University of Copenhagen, Department of Plant and Environmental Sciences, Section for Environmental Chemistry and Physics, Thorvaldsensvej 40, Frederiksberg C DK-1871, Denmark; ◆ Empa−Swiss Federal Laboratories for Materials Science and Technology, Technology and Society Laboratory, St. Gallen CH-9014, Switzerland

**Keywords:** fluoropolymer production, per- and polyfluoroalkyl substances, PFAS manufacturing, PFAS emissions, environmental monitoring

## Abstract

Fluoropolymers are widely used across sectors, but their production is associated with emissions of perfluoroalkyl and polyfluoroalkyl substances (PFASs), which are mobile, persistent, and toxic. In this work, we compiled a global inventory of fluoropolymer production plants (FPPs) and assembled PFAS concentration measurements for various media in their vicinity. We identified 52 currently operating FPPs across 11 countries and 41 cities. For 12 FPPs, in 12 different cities, there are peer-reviewed site-specific PFAS measurements specifically attributed to the FPP. At these 12 sites, at least 236 individual PFASs have been detected across multiple environmental media, including surface water, groundwater, air, dust, soils, sediments, plants, animals, and humans, with reported detections at distances of up to approximately 150 km from FPPs. Perfluoroalkyl carboxylic acids (PFCAs) and perfluoroalkyl ether carboxylic acids (PFECAs) were most frequently measured, often at concentrations two to three orders of magnitude higher than those measured in regions without nearby FPPs. Using high-resolution population data, we estimate that approximately 14 ± 2 million people (uncertainty reflecting ± 10 km uncertainty in facility locations) live within 10 km of an FPP. These people are potentially affected by FPP-associated contamination, with the largest population shares in China (≈52%), Japan (≈24%), Europe (≈13%), and the United States (≈9%). These regional proportions largely mirror differences in population density and the number of identified production facilities. This inventory reveals the large and complex global scale of PFAS contamination from fluoropolymer production, underscoring the need for expanded systematic monitoring and risk management efforts, including regulation.

## Graphical abstract

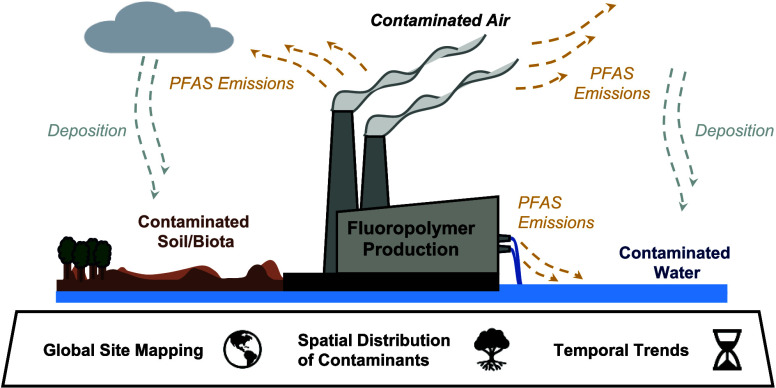

## 1 Introduction

Since the invention of polytetrafluoroethylene (PTFE) in 1938,^1,2^ the fluoropolymer industry has steadily grown to an estimated global production volume of 389 000 tonnes/yr and an estimated market worth of US$7.9 billion in 2023,^3^ projected to increase to 478 000 tonnes/yr (US$9.6 billion) by 2026.^3^ Fluoropolymers are defined as containing repeating units of carbon backbone chains with fluorine (F) atoms covalently bonded to the backbone carbon (C) atoms.^4^ Most fluoropolymers belong to the class of per- and polyfluoroalkyl substances (PFASs, as defined by the OECD as any chemical with at least one −CF_3_ or −CF_2_– group).^5^ The fluorinated carbon chain gives fluoropolymers outstanding resistance to water, oil, fire, most chemicals, and extreme temperatures.^6^ As a result, the dense fluorine coverage lowers surface energy, resulting in, e.g., nonstick and low-friction properties. These combined material properties make fluoropolymers highly durable and suitable for applications ranging from spacecraft components to nonstick frying pans.

However, the production (i.e., making the fluoropolymers, including monomer synthesis), manufacturing (i.e., transforming the raw material into finished goods), use, and disposal of fluoropolymers are associated with emissions of a wide range of organofluorine substances, including high-molecular-weight compounds such as fluoropolymer particles, but also low-molecular-weight nonpolymeric substances.^7^ Fluoropolymer production, in particular, is known to be associated with high volumes of emissions of fluorinated gases,^8^ perfluoroalkyl carboxylic acids (PFCAs),^9,10^ and perfluoroalkyl ether carboxylic acids (PFECAs),^11–13^ among others. The human health hazards of some of these PFASs, including those that are fluorosurfactant processing aids and production byproducts, have been well documented with clear links to thyroid disease, liver damage, increased cholesterol levels and lipid dysregulation, kidney and testicular cancer, and developmental impacts in unborn children.^14–16^

Emissions originate from each step of the process at a fluoropolymer production plant (FPP). First, monomer synthesis starts with the fluorination of small organic precursors with hydrofluoric acid (HF), followed by different types of reactions, including pyrolysis, to form the desired monomer. This process can lead to unintended fluorinated organic byproducts, including large volumes of fluorinated gas emissions. For example, the potent greenhouse gas trifluoromethane (HFC-23) is a byproduct formed in the production of chlorodifluoromethane (HCFC-22; used to make the monomer trifluoroethylene or TFE).^8,17^ During polymerization, incomplete and side reactions can result in byproducts such as oligomers and other low-molecular-weight fluorinated compounds, which have been increasingly detected around FPPs using nontargeted screening with high-resolution mass spectrometry.^18–21^ Unreacted monomers and processing aids, such as fluorosurfactants used to stabilize aqueous dispersions, may also be released into air or wastewater at the production site and during subsequent processing steps.^8,18,22^ Other emissions related to fluoropolymer production include those from fugitive gases and liquids from bulk storage, from the storage and treatment of waste materials (e.g., incineration of waste), and from cooling systems (fluorinated gases as refrigerants).^8^

Environmental contamination related to the use of fluorinated processing aids has historically received the most attention. The most widely used fluoropolymer processing aids were the ammonium salts of perfluorooctanoic acid (PFOA) and perfluorononanoic acid (PFNA), but due to concerns over their human health and environmental impacts, their use was eliminated by 2015 by eight major global fluoropolymer and fluorotelomer producers (Arkema, AGC, BASF, Clariant, Daikin, 3M/Dyneon, DuPont, and Solvay Solexis) under the United States (US) Environmental Protection Agency (EPA) 2010/15 PFOA Stewardship Program.^23^ However, the production, use, and therefore emissions of PFOA have largely shifted to Asia,^24,25^ including China and India.

In the European Union (EU) and the US, fluoropolymer producers have substituted PFOA and PFNA with structurally similar PFECAs, including, for example, the ammonium salt of hexafluoropropylene oxide dimer acid (HFPO-DA or GenX) by Chemours.^26^ However, HFPO-DA has also been shown to be toxic^27,28^ and has been measured in surface waters worldwide,^29^ thereby leading to concerns for human and ecosystem health.^30^ In addition to fluorinated alternatives such as PFECAs, some manufacturers have developed nonfluorinated surfactants as processing aids.^31,32^ However, recent studies indicate that these can still lead to the formation of even greater quantities of unintended fluorinated byproducts, including PFASs and non-PFASs.^33^ Moreover, eliminating emissions from processing aids will not eliminate total emissions from fluoropolymer production as significant emissions result from other stages of the process as described above.

In response to growing concerns related to the high persistence and other hazardous traits of PFASs, Denmark, Germany, The Netherlands, Sweden, and Norway submitted a restriction proposal (“uPFAS”) in 2023 to universally restrict PFASs (specifically to restrict them from being “manufactured, used, or placed on the market as substances on their own”) under the EU’s chemicals regulation, REACH (Registration, Evaluation, Authorization and Restriction of Chemicals).^34^ This proposal treats PFASs as a single class of chemicals to be managed, which would prevent the continued practice of substituting one harmful PFAS (e.g., PFOA) with another (e.g., HFPO-DA), a practice known as “regrettable substitution.” Notably, the updated uPFAS background document proposes a derogation for PFAS manufacturing (which includes fluoropolymer production as defined in this paper), for which manufacture (production) would be allowed to continue with specific average emission limits for an unlimited time,^35^ assuming abatement techniques would be effective to limit emissions without limiting production volumes. The restriction proposal is currently under evaluation by the scientific committees to the European Chemicals Agency (ECHA), who will provide their scientific opinions to the European Commission in 2026.^36^

However, to properly understand the environmental and human health implications of fluoropolymers, a more complete picture of PFAS emissions associated with their lifecycle is essential. While studies on PFAS emissions associated with individual FPPs in one or more media are increasing, a global synthesis and overview remain missing, including both the locations and the diversity and levels of measured PFASs.

As such, in this study, we have three main objectives: 1) to identify, locate, and document FPPs worldwide – as a foundational step in assessing potential PFAS contamination and exposure risks; 2) to compile PFAS measurements in environmental media surrounding these FPPs worldwide; and 3) to estimate how many people globally may be affected by local FPP contamination to illustrate the potential human exposure, and therefore public health risk. The latter two objectives aim not only to assess the magnitude of PFAS emissions associated with fluoropolymer production to inform regulatory decision-making but also to identify and highlight critical knowledge and data gaps to guide targeted future research.

## 2 Methods

### 2.1 Global Mapping of Fluoropolymer Production Plants

The initial list of major FPPs was compiled from a combination of publicly available and proprietary sources. Foundational information was drawn from earlier work by Armitage et al.^37^ and subsequent investigations by Wang et al.^10^ Additional information on facility locations, operating companies, and produced fluoropolymer types was obtained from industry market research reports,^38,39^ company-specific documentation, peer-reviewed literature, government documents, market report summaries, and news articles. The FPP list presented in Annex A^40^ of the updated uPFAS background document as well as the recent fluoropolymer OECD report^7^ were also consulted. GPS coordinates of each FPP were determined using *Google Maps* in satellite view; for more details on this method, see Supporting Information (SI) Section S1a.

Note that our scope is specifically limited to plants which produce fluoropolymers, i.e., where polymerization of monomers leads to polymers with fluorinated backbones. Plants that only produce side-chain fluorinated polymers, other PFASs, or which only perform downstream processing of fluoropolymers are *not* included in this study. However, note that terminology is not always consistent in the literature, so FPPs (per our definition) may also be referred to in the literature and documentation as fluoropolymer manufacturing facilities, factories, sites, or plants, or, less precisely, as fluorochemical manufacturing or production facilities, plants, or sites. It was thus essential to verify the activities of each plant, as informed by our sources, before including them in our study. Here, only sites where polymerization occurs (which we call fluoropolymer production), as informed by our sources, were included. The complete list of FPPs and associated information is provided in SI Table S1.

### 2.2 Literature Search of PFAS Measurement Data

To collect data on PFAS measurements near FPPs, we searched extensively through the peer-reviewed literature with a two-step approach. No literature more recent than September 20, 2025, was included.

The initial body of literature was compiled by coauthors based on studies that they were aware of. To supplement this initial list, we conducted a literature search using *Scopus* and *Google Scholar.* In *Scopus,* we searched *[(“fluoropolymer” OR “fluorochemical”) AND (“manufacturing” OR “production”) AND (“site” OR “facility” OR “plant”)]* and screened the 270 results by title and then evaluated them by content, as detailed in the next paragraph. In *Google Scholar,* the same search string produced over 23 000 results, which was too many to screen. Therefore, we used the two following search strings: *[“fluoropolymer production plant” OR “fluoropolymer production site” OR “fluoropolymer production facility” OR “fluoropolymer manufacturing site” OR “fluoropolymer manufacturing plant” OR “fluoropolymer manufacturing facility” AND “measurement”*] and *[“fluorochemical production plant” OR “fluorochemical production site” OR “fluorochemical production facility” OR “fluorochemical manufacturing site” OR “fluorochemical manufacturing plant” OR “fluorochemical manufacturing facility” AND “measurement”*], each producing around 400 results, which again were screened first only by title and then later by content. Furthermore, for each known FPP location, we searched *Google Scholar* with *[(“fluorochemical” OR “fluoropolymer”) AND (“production” OR “manufacturing”) AND “measurement”]* and the location name, e.g., the country (*AND “Japan*”) or the state (*AND “Texas*”), then only the first ∼100 search results were screened. After these searches, the references within the relevant literature were examined for additional relevant sources that may not have surfaced in the database searches.

Studies were included if they met all three of the following criteria: a) PFAS concentrations were measured in any medium near an FPP; b) PFAS measurements were attributed to that FPP, according to the authors of the study; and c) the measurements were reported in the peer-reviewed literature in English.

Studies were excluded if they met at least one of the following criteria: a) PFAS measurements were not reported near an FPP; b) sampling years were not reported;^41–43^ c) specific locations/distances or location/distance ranges were not reported;^20,44^ d) the measurements were not quantitative (i.e., they could not provide any concentrations, only peak areas);^12,13,18,21,45^ e) samples were from treated drinking water; or f) blood samples were from individuals with known/potential occupational exposure.^46,47^

Furthermore, for each FPP where we found concentration measurement data according to our criteria above, we also evaluated the presence of other potential PFAS-emitting activities in the surrounding area. Using facility coordinates, each FPP location was inspected in *Google Maps* (satellite view) to identify neighboring industrial sites and company names within the same or adjacent industrial zones or within a 10-km radius. Company names and activities were then checked using publicly available sources (e.g., company websites) to determine whether any nearby facilities were known or suspected PFAS producers, processors, or users. For each FPP site, we classified the presence of nearby PFAS-related activity in Table S1 as “Yes”, “Maybe”, or “No.” “No” indicates that no other chemical production or PFAS-related activity was visible or reported; “Maybe” indicates nearby chemical industries with no confirmed PFAS production but possible PFAS-related activity (e.g., use); and “Yes” indicates a dense cluster of documented PFAS production or use in the vicinity. Based on this analysis, measurements from two Chinese sites (Fuxin^25,48–51^ and Changshu^25,52–56^ ) were excluded from our inventory because there is high confidence that there is other non-FPP-related PFAS production nearby, meaning that the PFAS concentration levels cannot be uniquely attributed to the FPPs.

### 2.3 PFAS Measurement Data Collection

For each included study, we collected every reported PFAS, its measured concentration in each sample (or “n.d.” if it was measured but not detected), the sampled medium, the year of sampling, the distance from the sampling location to the FPP, and, for river water and sediment samples, whether it was upstream or downstream of the FPP. Where possible, data were taken directly from the text or tables in the publication. If concentration data were not directly available (e.g., if only summary statistics were provided or if data were only shown in figures), raw data were requested from the corresponding author. We were provided with raw data for three publications.^11,57,58^ If data could still not be obtained this way, we used the summary statistics as data points and/or extracted data from figures (see SI Section 1b for this method).

For each sample, the PFAS_sum_ was calculated (if not already provided by the authors), and the concentration of each reported PFAS was summed. Different studies report different numbers of PFASs, so the PFAS_sum_ does not consistently represent the same set of PFASs. Indeed, for three studies,^59–61^ only PFOA was reported, so the PFAS_sum_ reflects only PFOA concentrations. Furthermore, a PFAS_sum_ could not be calculated for three studies^62–64^ for which data were extracted from figures because individual samples could not be determined.

The distances between the sampling locations and the FPP were sometimes provided directly, but were otherwise: a) estimated graphically from a map provided in the paper, if a scale was also provided, or b) calculated using given GPS coordinates of the sampling locations and the GPS location of the FPP. Distances were always calculated as straight-line distances, without accounting for migration routes. For surface water and sediment samples, sampling locations upstream and downstream of the FPP were additionally distinguished. Note that for human blood samples and pet samples, the distance represents the straight-line distance between the person’s residential address and the FPP.

### 2.4 Organization of the Collected Measurement Data

A large variety of media have been sampled and measured for PFASs. To better organize the data collected, we grouped them into six medium categories:

1. *Surface water*, including samples taken from river or streamwater,^19,20,22,24,25,65–85^ seawater,^83,86,87^ effluent channels,^19,66,68^ and other waterways, lagoons, ponds, or channels.^19,20,76^

2. *Groundwater*, including samples collected from private or monitoring wells,^19,41,57,58,69,73,88–91^ and groundwater from other sampling means.^19,75,92,93^

3. *Air*, including measurements of the gas phase,^94,95^ of airborne particulate matter (aerosol),^11,68,94–99^ or of both combined.^59,96,100^

4. *Dust*, including dust samples collected from surfaces inside homes^58,101^ and from outside surfaces.^64,94,101,102^

5. *Soil and sediment* , including samples of river sediment,^22,66,67,74,77,78,86,103,104^ sea sediment,^83,87^ and soils.^60,62,71,93,100,102,105–109^

6. *Biological samples* with three subcategories of a) *plants* including samples of various plant leaves,^108,110^ plant roots,^67,108^ plant shoots,^67^ pine needles,^111^ and various edible and nonedible parts of fruit, vegetable, and grain crops;^106,112^ b) *blood serum* including samples of human blood serum,^57,61,62,65,106,113–117^ fish blood serum,^65^ dog and horse blood serum,^89^ and alligator blood serum;^118^ and c) *animal tissue* including fish fillet,^74^ otter liver,^63^ carp liver and muscle,^65^ seabird organs,^119,120^ bird eggs,^121,122^ and homogenized earthworms^108^ and various aquatic organisms.^86^

Furthermore, to organize the large number of PFASs measured, we grouped them into four main categories of PFAS types: PFCAs, perfluoroalkanesulfonic acids (PFSAs), PFECAs, and “others”; the complete list of categories and which substances are included in them is provided in Table S2.

All measurement data that we collected as described above are tabulated with corresponding references into a spreadsheet publicly available in Table S3. Note that individual sample data from the GenX Exposure Study^123^ are excluded from this public file due to human subjects’ protections of these data; however, summary statistics published by Kotlarz et al.^57^ and Proctor et al.^58^ are provided.

### 2.5 Potentially Exposed Population Calculations

We estimated populations living near each FPP using the Global Human Settlement Layer (GHSL) 2020 population grid (P2023A release, approximately 100 m resolution) within Google Earth Engine.^124^ Circular buffers were drawn at distances from 5 to 50 km around each FPP in 5 km increments, and population totals were calculated by summing the GHSL pixels within each buffer. Estimates at 10 and 50 km were extracted for further analysis. To address coordinate uncertainty, we repeated calculations across 10 randomized realizations of each FPP location with variation within a 10 km radius. We also applied an overlap adjustment method to avoid double-counting populations in areas where FPP buffers intersect. Further methodological details, including population grid handling, uncertainty quantification, and validation against independent sources, are provided in SI Section S1c.

## 3 Results and Discussion

### 3.1 Confirmed Fluoropolymer Production Plants Worldwide

A total of 56 FPPs were identified globally and distributed across 11 countries. The locations of the identified FPPs are visualized in Figure 1. The compiled list of FPPs, along with their GPS locations, street addresses, facility names, produced fluoropolymer types, operational status, confidence levels regarding nearby PFAS producers, and associated references, is provided in Table S1. The identified FPPs encompass three historical facilities (red crosses in Figure 1: Zakłady Azotowe in Tarnów, Poland, produced PTFE until the 1990s; AGC Chemicals in Bayonne, NJ, USA, produced PTFE until 2007; Hindustan Fluorocarbons in Rudraram, India, produced PTFE until 2021) and one FPP currently under construction (Synesqo (formerly Solvay Specialty Polymers) in Augusta, GA, USA), which was excluded from the visualization in Figure 1. This distribution highlights the concentration of fluoropolymer production capacity in established industrial regions. While many facilities are concentrated in the USA and Europe, the largest nearby populations are in China and Japan, reflecting high population densities around several plants. These FPPs are involved in the production of high-molecular-weight fluoropolymers, such as polytetrafluoroethylene (PTFE), fluorinated ethylene-propylene (FEP), perfluoroalkoxy alkane (PFA), ethylene tetrafluoroethylene (ETFE), polyvinylidene fluoride (PVDF), polychlorotrifluoroethylene (PCTFE), ethylene chlorotrifluoroethylene (ECTFE), polyvinyl fluoride (PVF), fluoroelastomers, and others. The facilities are operated by a mix of large multinational producers, including Chemours, Daikin, AGC, Solvay/Syensqo, Arkema, Gujarat Fluorochemicals, and Central Glass, as well as several major regional manufacturers, particularly in China (e.g., Dongyue Group, Shanghai 3F, Juhua, Meilan Chemical Group) and Russia (HaloPolymer). Based on market share estimates reported in the EU PFAS restriction proposal background document,^40^ Shandong Dongyue Group is currently the largest fluoropolymer producer (approximately 13%, followed by Chemours (approximately 12%), Daikin (approximately 11%), and Solvay (approximately 8%). Importantly, the fluoropolymer industry is dynamic: mergers, expansions, and facility closures are common, and at least one major producer has recently announced to exit fluoropolymer production (3M by the end of 2025).^125^ As a result, the number and distribution of FPPs are not fixed, and our data set reflects a time-specific snapshot of known operations as of October 2025. While we utilized multiple industry and regulatory sources for our mapping, we do not claim that this is a completely comprehensive list, as information is not always publicly available or consistent across all regions.

**Figure 1:**
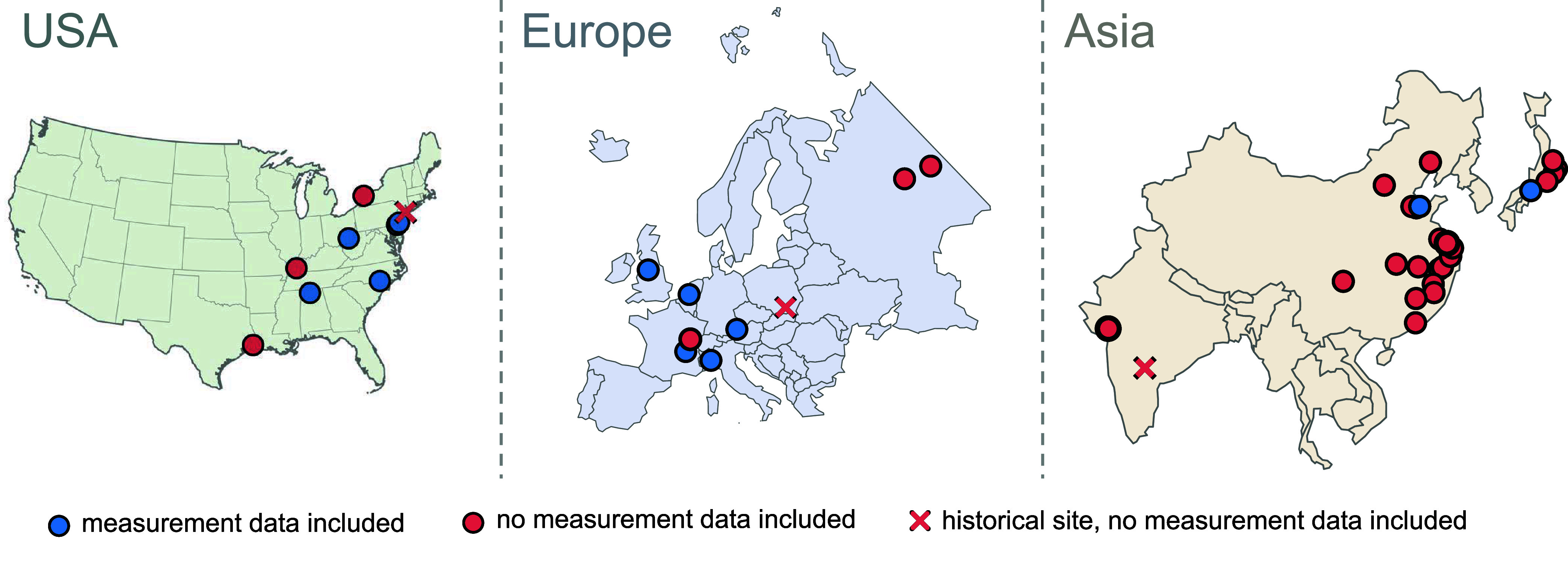
Spatial distribution of identified fluoropolymer production plants (FPPs) compiled in this study, with the USA (green), Europe (blue), and Asia (yellow). Symbols: blue dots = sites with measurement data included in our inventory in Table S3, red dots = sites without measurement data included in Table S3, and red crosses = historical sites without measurement data included in Table S3; see Methods section for details.

### 3.2 Data Overview and Structure

We compiled PFAS measurement data in the vicinity of 12 FPP locations (blue dots in Figure 1) by screening the peer-reviewed literature (see the Methods section). Figure 2 visualizes the data structure as an alluvial diagram. Flow width represents the number of data points (not concentration-weighted), and flows are colored and grouped by sampling medium. In total, 73 references contributed data spanning 2000–2024 (25 distinct years). Reference coverage by site was highest at Huantai County (22 references), Fayetteville (17), Parkersburg (7), and Pierre-Bénite (6), followed by Dordrecht (5); Spinetta (4); Osaka and Thornton-Cleveleys (3 each); Burgkirchen and Thorofare (2 each); and Decatur and Deepwater (1 each). Nondetects were excluded prior to visualization to focus on quantifiable PFAS concentrations. In addition to nondetects (n = 27 927), the data set comprised 41 666 valid concentration records out of a total of 69 593 entries. The six medium groups defined in the Methods section are altered to make five groups for visual clarity here: water (n = 8815, including both groundwater and surface water), air (n = 2369), biological samples (n = 5266), soil and sediment (n = 21 594), and dust (n = 3622).

**Figure 2:**
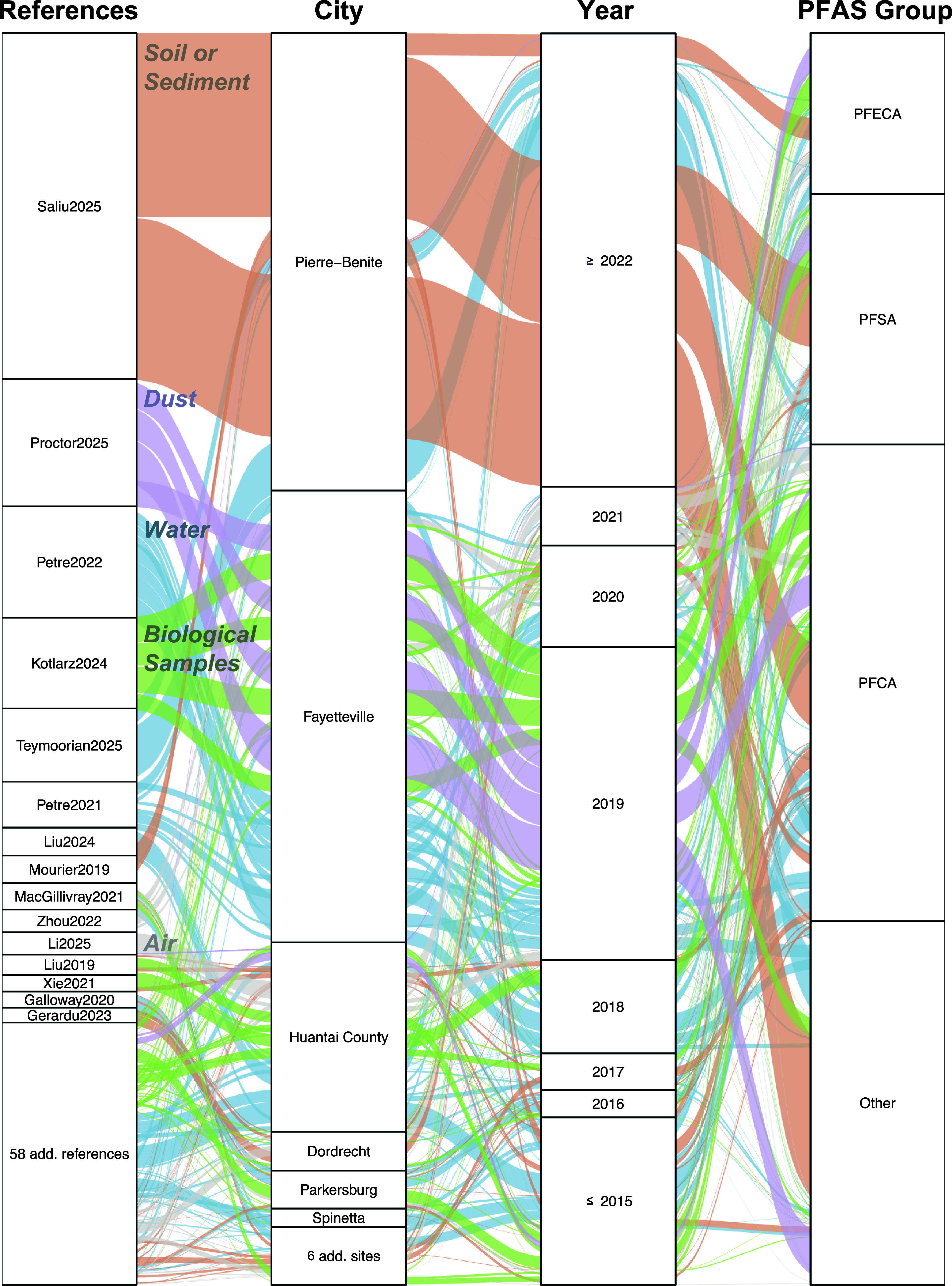
Alluvial diagram summarizing the structure of compiled PFAS measurement data around fluoropolymer production plants (FPPs). From left to right, flows connect (i) the reference source, (ii) the city of the corresponding FPP, (iii) the sampling year, and (iv) the PFAS group analyzed. Flow width is the number of individual data points. Colors indicate the environmental medium in which PFASs were measured: water (blue), air (gray), biological samples (green), dust (purple), and soil or sediment (rose). In total, 73 references contributed 57 159 valid concentration records (not including nondetects) spanning 2000–2024, linked to 12 sites in North America, Europe, and Asia.

Analytes are classified into four PFAS groups according to their functional headgroup chemistry. Perfluoroalkyl carboxylic acids (PFCAs; n = 15 241) are dominated by perfluorooctanoic acid (PFOA; n = 2389), perfluorononanoic acid (PFNA; n = 1714), and perfluoroheptanoic acid (PFHpA; n = 1632). Perfluoroalkanesulfonic acids (PFSAs; n = 6412) primarily comprised perfluorooctanesulfonic acid (PFOS; n = 1704), perfluorohexanesulfonic acid (PFHxS; n = 1099), and perfluorobutanesulfonic acid (PFBS; n = 943). Perfluoroalkyl ether carboxylic acids (PFECAs; n = 5214) included ether-containing compounds such as PFO4DA (n = 321), PMPA (n = 557), and hexafluoropropylene oxide dimer acid (HFPO-DA, “GenX”; n = 1121). The Other group (n = 14 799) consisted mainly of fluorotelomer- and sulfonamide-based PFASs, including 6:2 fluorotelomer sulfonates (6:2 FTS; n = 443), 4:2 FTS (n = 228), 8:2 FTS (n = 230), and perfluorobutane sulfonamide (FBSA; n = 367). The complete analyte-to-group mapping is provided in Table S2. Note that these statistics only reveal how many times each of these substances was measured, not how high the measured concentrations were (see next section).

Figure 2 highlights that water samples dominate most site–year combinations and that recent campaigns (2018–2023) contributed the bulk of PFCA and PFECA observations. However, the data availability is highly uneven across FPPs: only a handful of sites (most notably Fayetteville, Huantai County, and Pierre-Bénite) have been studied in sufficient detail to allow for temporal and cross-media comparisons, while many other FPPs are represented by only one or two campaigns or have no peer-reviewed literature at all.

### 3.3 Measured PFAS Around Fluoropolymer Production Plants

Across all media in the compiled data set, a total of 236 distinct PFASs were measured. These include PFCAs, PFECAs, PFSAs, perfluoroalkyl ether sulfonic acids (PFESAs), fluorotelomer carboxylic acids (FTCAs), fluorotelomer sulfonic acids (FTSAs), fluorotelomer alcohols (FTOHs), and other PFASs with phosphate, phosphonic acid, sulfonamide, sulfonamidoacetic acid, sulfonamidoethanol, or other functional groups (all individual PFASs are listed in Table S2 with CAS numbers and references; all substances are additionally shown for each sampling medium in Figures S14–S21). Figure 3 shows the seven PFASs with the highest measured concentrations in surface water since 2018 for the five sites with the most surface water measurement data. At all five sites, the PFASs with the highest concentrations are almost entirely PFCAs and PFECAs, with the exception of fluorotelomer contamination at Pierre-Bénite. Each site has a distinct profile of dominant PFASs, for example, at Huantai County and Thornton–Cleveleys, PFOA is predominant, whereas at Burgkirchen and Fayetteville, HFPO-DA is most dominant.

**Figure 3:**
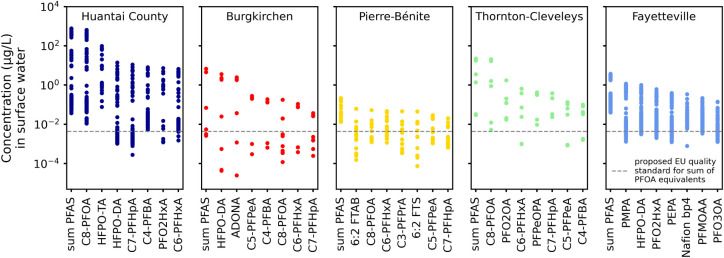
Concentrations of PFAS_sum_ and the seven highest-concentrated substances measured in surface waters near five of the FPPs with the most surface water data (Huantai County, China; Burgkirchen, Germany; Pierre-Bénite, France; Thornton–Cleveleys, England; Fayetteville, NC). Only measurements from 2018 or later are shown. The gray dashed line at 0.0044 μg/L represents the proposed EU environmental quality standard for the sum of PFOA equivalents of a specific set of PFASs in surface water.^126^ See Table S2 for the list of substances’ full names and Table S3 for the data sources.

Because of this heterogeneity, we use PFAS_sum_ for comparing contamination across different sites and times. PFAS_sum_ values at the sites shown in Figure 3 are typically in the range of 0.001 to 10 μg/L, with a minimum of 0.003 μg/L (downstream from the FPP at Burgkirchen in 2018)^25^ and a maximum of 777 μg/L (downstream of the FPP in Huantai County in 2019).^70^ However, note that PFAS_sum_ does not represent the same set of analytes for each study. Some studies only measured PFOA, whereas others measured more than 80 different PFASs. Consequently, PFAS_sum_ represents the total concentration of PFASs actually measured in a study, but must be interpreted as a *minimum* total possible PFAS concentration in the environment, as no study captures all potential PFASs. Comparisons between sites should therefore be made with caution.

We thus evaluated trends in (i) PFAS_sum_ concentrations as a function of distance from the facility, (ii) concentrations in comparison with other PFAS sources and regulatory benchmarks, and (iii) potential population exposure to PFASs associated with fluoropolymer production. We also assessed PFAS concentrations as a function of time, but no strong conclusions could be made due to a lack of data consistency and comparability across time; see SI Section S2 and Figures S2–S8 for this analysis. The data set also supports more detailed investigations into individual substances, sites, years, and mediums. To facilitate further research, the complete data set is provided in SI Table S3.

#### 3.3.1 Concentrations with Distance from Facility

After removing nondetects (n = 27 927), 40 375 records (96.9%) included concentrations with information on the distance to the nearest FPP, while 1291 records (3.1%) did not. Distances were typically short: 0.1–5 km (n = 15 411) was the single largest distance bin, followed by 5–10 km (n = 7625), 10–25 km (n = 9304), and 25–50 km (n = 2273). A smaller number of measurements were made at 50–100 km (n = 2839) and beyond 100 km (n = 2260) from FPP sites. Effluent or near-source samples (≤0.1 km) accounted for 663 measurements.

Figure 4 shows the PFAS_sum_ concentrations around FPPs as a function of distance. Measurement data are grouped according to six sampling media: surface water, groundwater, air, dust, soil/sediment, and biological samples (detailed in the Methods section). Within each medium, data points are colored according to their associated FPP. Analogous plots for individual PFAS groups (PFCA, PFECA, PFSA, PFOA, and HFPO-DA/GenX) are provided in SI Figures S9–S14.

**Figure 4:**
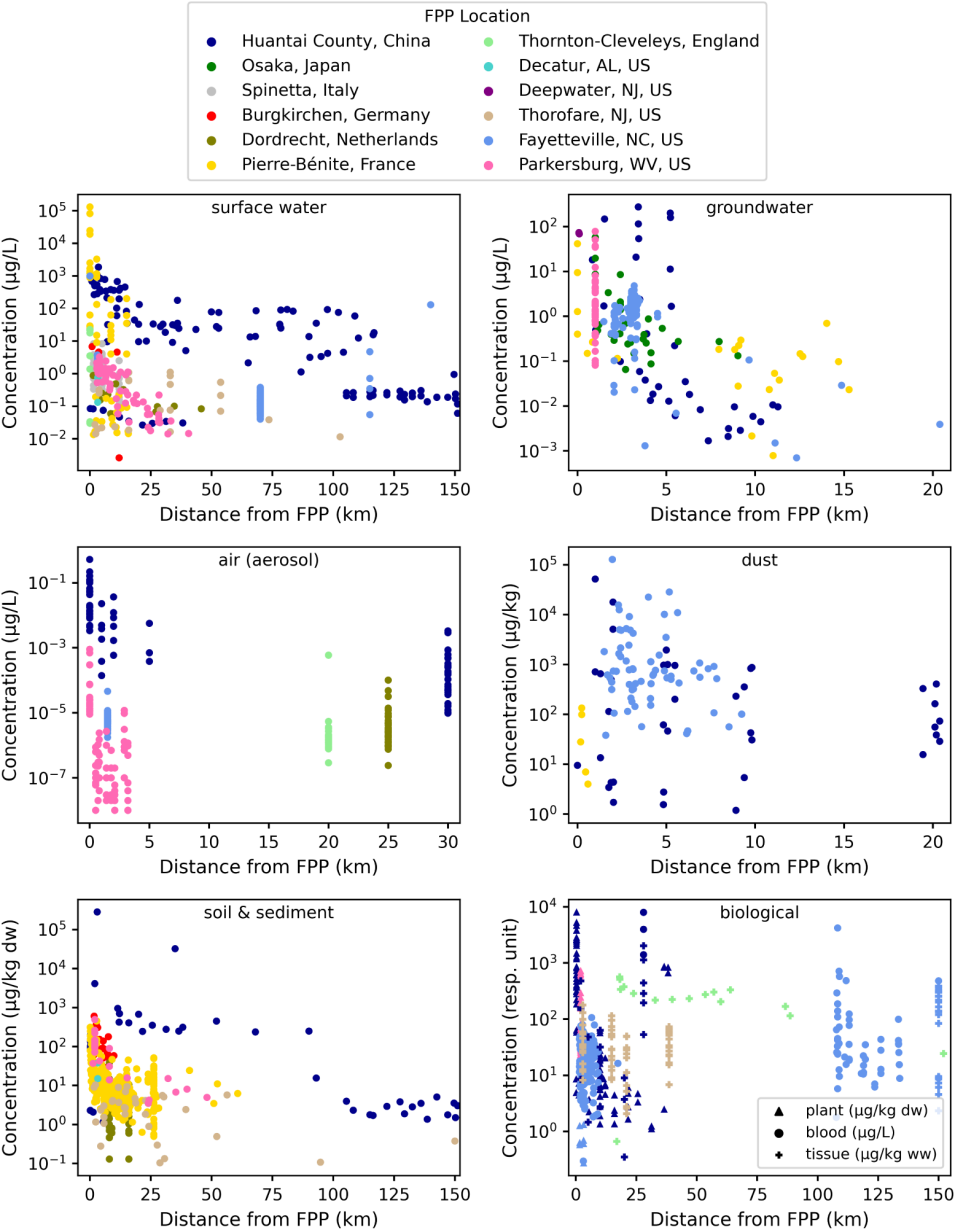
PFAS_sum_ values measured at varying distances (km) from a fluoropolymer production plant (FPP), according to sampling media. Colors refer to the location of the FPP; note that actual sampling locations may be different depending on how far away the sampling was from the FPP. For surface water and sediment measurements, only downstream measurements are plotted for simplicity. Measurements of biological samples include plant tissue (roots, shoots, leaves, fruits, vegetables, and grains), blood serum (of humans, dogs, horses, seabirds, fish, and alligators), and animal tissue (of bird organs, fish organs, chicken eggs, and fish fillet). Only dry weight (dw) concentrations are plotted for soil/sediment and plant measurements; only wet weight (ww) concentrations are plotted for animal tissue; and only particulate phase (aerosol) measurements are plotted for air. All measurements have been previously reported; see Table S3 for the complete table of data and sources.

In general, the highest concentrations were measured at or close to facility coordinates (i.e., distance <1 km). This trend was apparent in most media, including surface water (reaching up to 100 000 μg/L), groundwater (up to 300 μg/L), air (up to 0.5 μg/L), dust (up to 100 000 μg/kg dry weight (dw)), soil/sediment (up to 280 000 μg/kg dw), plants (up to 8000 μg/kg dw), and blood serum (up to 8000 μg/L). Overall, concentrations decrease with increasing distance, most clearly in surface water (Huantai County, Pierre-Bénite, Parkersburg, Thorofare), groundwater (Huantai County, Fayetteville, Osaka), and dust (Huantai County, Fayetteville). Similar distance-related declines are also evident in soil/sediment (Huantai County, Parkersburg, Burgkirchen, Thorofare) and plants (Huantai County).

The variability in the concentration at similar distances is a notable feature of Figure 4. Medium-specific processes may explain much of this spread. In water, river flow and effluent variability strongly influence concentrations, with higher flow rates generally causing dilution. In air and dust, the wind direction, wind speed, precipitation, and emission variability determine dispersion, while sampling upwind vs downwind can yield large differences even at the same nominal distance.^58,97,101^ Since air samples are usually at least multiple-hour composite samples, and since wind direction and weather can be highly variable within this time frame, it becomes difficult to correct for these influences (as compared to, e.g., the more consistent nature of upstream and downstream influences in river water). In soils and sediments, on the other hand, proximity to water bodies and effluent emissions, as well as sampling depth, affect measured concentrations.^60,103^ In plants, uptake differs across species and between tissues of the same plant.^67,106,110^ In animals, aside from differences between different organs, there are both inter- and intraspecies differences in physiology (e.g., sex, age)^46,116,127,128^ and behavior (e.g., feeding and flight ranges)^119^ that contribute to variation.

For all media, the variability in the extraction efficiency (recovery) of the analytical methods and the variations due to sampling designs can also strongly influence the reported levels. Furthermore, the lack of harmonized quality-assurance and quality-control (QA/QC) protocols across the diverse set of included studies introduces additional uncertainty, as differences in analytical sensitivity and quantification methods can affect the comparability of the results. Moreover, variability also reflects random emission patterns, varying production history, methodological differences (including especially the number and type of PFASs targeted), and inherent intersample variation. Once released, PFASs are highly mobile in air and water and persistent, and they partition into air, water, soil, and biological samples, ensuring eventual detection across multiple media. The complexity of these interconnected flows and uptake processes, combined with diverse sampling conditions, helps explain the broad ranges observed in Figure 4.

#### 3.3.2 Comparison to Other Sources and Benchmarks

PFAS concentrations observed near FPPs are high in both absolute terms and relative to those of other known sources. For example, individual PFAS concentrations measured in surface waters within 10 km of FPPs were frequently above 1 μg/L, with maxima exceeding 1000 μg/L (Figures S9 – S14). These levels are similar to those reported at AFFF-impacted sites, such as airports and military bases,^129–131^ which are widely recognized as PFAS hotspots. By contrast, concentrations of PFOA in rivers and lakes in the United States without known point sources typically remain below 0.01 μg/L.^132^ Similarly, soils in the immediate vicinity of FPPs often contain individual PFASs in the 1–10 μg/kg range, with hotspots exceeding 100 μg/kg (Figures S8–S14). These values are generally lower than the extreme PFOS concentrations (up to >300,000 μg/kg) reported at AFFF fire training areas but fall within the range of PFOA concentrations observed at such sites.^133^ Moreover, PFAS levels near FPPs are similar to soil burdens reported at secondary-source sites impacted by land application of biosolids or industrial wastes, underscoring the significance of FPPs as emission sources that create PFAS hotspots.^133^

For comparing PFAS concentrations near these FPPs to a regulatory standard, we can look to the EU Water Framework Directive’s proposed quality standard for PFASs in surface and groundwater, which is set to be 0.004 μg/L for the sum of PFOA equivalents for a specific set of 25 PFASs (including PFOA, PFOS, HFPO-DA, and 22 others).^126^ For PFOA-equivalent concentrations, the substance concentration is multiplied by its relative potency factor (between 0.001 and 10), as defined in the Water Framework Directive. Because our data inventory is a heterogeneous set of 73 different studies, each measuring a variety of types and sets of substances, it is not possible to calculate a consistent sum of PFOA equivalents for each sample, hampering intercomparison. Still, we show the concentrations of the seven highest measured substances in surface water for five sites in Figure 3, along with the proposed 0.004 μg/L quality standard, in order to illustrate the relative orders of magnitude. Even PFOA concentrations since 2018 at four of these sites are higher than the PFOA-equivalent quality standard, indicating that total contamination is already *at least* greater than the proposed water quality standard, even in recent years.

#### 3.3.3 Potential Population Exposure

Although concentrations generally declined with increasing distance, PFAS_sum_ remained consistently elevated at substantial ranges, typically up to 20 km in air, dust, and plants, and sometimes even beyond 100 km in surface water, soil/sediment, and animals (Figure 4). However, at such distances, attribution to a specific facility becomes increasingly uncertain, as additional regional or diffuse sources may contribute. Nevertheless, given the high mobility in air and water and the persistence of PFASs, including their detection in remote regions,^134–136^ contamination from fluoropolymer production extends well beyond the maximum distances referenced here.

To translate these spatial patterns into potential population exposure estimates, we applied two distance bands around all confirmed FPPs. A 10 km radius represents a conservative zone of high likelihood of exposure to elevated PFAS concentrations, based on the near-field data discussed above. A 50 km radius captures a broader region where elevated PFAS contamination has been observed in some media but where contributions from other sources may also occur. Using these zones together with GHSL 2020 population data (100 m resolution), we estimate that 14 ± 2 million people live within 10 km of an FPP after adjusting for zone overlap (unadjusted total = 22.0 million). For a 50 km radius, the estimated population increases to 180 ± 10 million (unadjusted = 280 million), reflecting approximately 2% of the world’s total population. Regional shares of the 10 km (and 50 km) totals are China 52% (59% for 50 km), Japan 24% (20%), Europe 13% (10%), and the United States 9% (8%). These values illustrate the potential range of population exposure across distances; full distance-resolved results are provided in Figure S1. Uncertainty reflects imprecision in site-location data, as detailed in the Methods section.

## 4 Environmental Implications

This global inventory has key implications for the assessment of environmental pollution, regulatory action, and public health protection. At all sites with available PFAS measurements, contamination was found across environmental media, including surface water, groundwater, air, soil, dust, plants, humans, and animals, often extending tens of kilometers from the FPPs. Given the consistency of these patterns, it is reasonable to expect that other FPPs, currently lacking site-specific data, may show similar contamination if investigated. This inventory of measurements, therefore, highlights the large global scale of PFAS contamination associated with fluoropolymer production.

Furthermore, we estimate that 14 ± 2 million people live within 10 km of an FPP, and 180 ± 10 million live within 50 km, highlighting the scale of potential human exposure. Notably, most of the potentially exposed populations are in countries with little or no measurement data in the literature. Expanding monitoring and health assessments in these regions is critical given the extensive evidence of PFAS-associated health risks. Environmental monitoring of sediment, soils, and biota as well as wastewater sediment sludge used as fertilizers is important to assess potential uptakes in the terrestrial and aquatic food chains. Human biomonitoring programs (e.g., including blood, urine, or other relevant matrices) should also be prioritized near all facilities to provide insight into population-level exposures and potential health implications.^137^ To ensure comparability across data, sampling and monitoring methods should ideally be harmonized. Until this happens, the sampling and analysis should be reported with sufficient metadata to allow for future corrections of bias across the data sets. Although the current state of knowledge is already sufficient to encourage regulatory controls, more monitoring is essential to detect trends over time, distinguish between legacy and current emissions, and verify the effectiveness of risk management measures.

This inventory also highlights a measurement bias: most PFAS monitoring around FPPs has focused on PFCAs, PFECAs, and other ionic PFASs. Despite the recent paper by Dalmijn et al.^8^ showing high emissions of other fluorinated substances from FPPs, few studies^138^ have focused on measuring fluorinated gases, feedstock chemicals, oligomers, or polymers in direct connection to fluoropolymer production. Furthermore, nontargeted analysis of other PFAS types is equally important, as several studies using suspect screening have revealed additional PFAS types and polymerization-related compounds.^19,21,68,139^ Expanding suspect screening, as well as “Total PFAS” analysis near FPPs, particularly those employing nonfluorinated processing aids, is essential to quantify unidentified emissions and support future monitoring efforts. While industry claims that PFAS emissions originating from FPPs can be controlled in so-called “closed-loops,” ^32,140,141^ emission controls cannot be verified, managed, assessed, or enforced if emissions cannot be detected. Additional analytical standards and validated methods are needed to enable the reliable quantification of PFAS releases. Proactive regulation of recently identified substances is also needed so that the exposure and ecological risks posed by legacy PFASs are not repeated.

In sum, these findings demonstrate consistent patterns of environmental contamination around all FPPs where adequate data exist and support a more preventative, globally coordinated approach: prioritizing prevention of fluoropolymer production and non-PFAS alternatives; expanding monitoring methods for a diverse array of PFASs in air, soil, sediment, and biota near FPPs; expanding regulatory coverage to include more PFASs in vertical (sector, product) legislations, reducing fluoropolymer use, enhancing risk management efforts, promoting public information, and requiring greater transparency and accountability from industry.

## Supplementary Material





## Notes

Table S1: List of all fluoropolymer production plants. Table S2: PFAS full names, abbreviations, and their assigned groups. Table S3: All compiled concentration data. Table S4: Reference codes from Figure 2 and Tables S1–S3 with full citations (XLSX)
Extended methods and extra figures of PFAS concentrations in all media (PDF)

## ▶

University of Southern Denmark, Department of Green Technology & Danish Institute for Advanced Study, 5230 Odense, Denmark

## &

Energy & Process Systems Engineering, Department of Mechanical and Process Engineering, ETH Zurich, Tannenstrasse 3, 8092 Zurich, Switzerland.

## ††

Section Industrial Chemicals, Swiss Federal Office for the Environment, Bern 3011, Switzerland.
