# Correction to “Global Inventory of Fluoropolymer Production Plants and Their Associated PFAS Environmental Contamination”

**DOI:** 10.1021/acs.est.6c12429

**Published:** 2026-08-29

**Authors:** Anna J. Miller, Kevin Kleemann, Juliane Glüge, Rainer Lohmann, Ian T. Cousins, Dorte Herzke, Mark F. Miller, Amanda Rensmo, Xenia Trier, Zhanyun Wang, Martin Scheringer

In the original publication, most of the PFAS concentrations in air (aerosol) reported in micrograms per liter are 1000 times too high due to a unit conversion error from converting picograms per cubic meter to micrograms per liter.

Here, Figure 1 is the corrected version of Figure 3 of the original publication. The PFAS_sum_ concentrations in air (aerosol) are the only data affected. Figures S1–S13 and S14 in the Supporting Information of this correction are the corrected versions of Figures S2–S14 and S17, respectively, of the original publication.

**Figure 1:**
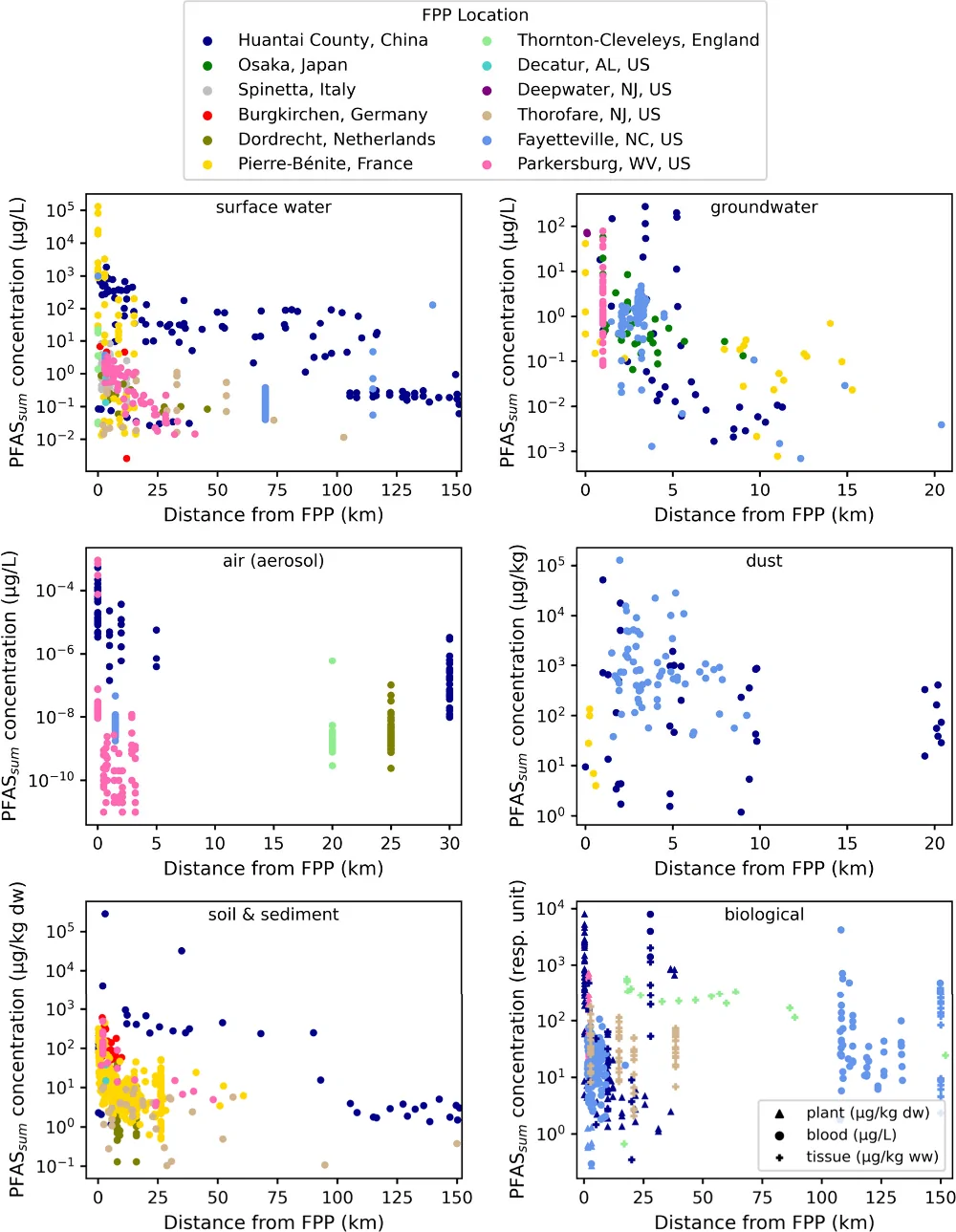
Corrected version of original Figure 3.

Table S1 in the Supporting Information of this correction is the corrected version of Table S3 of the original publication. Only the data that were originally reported in picograms per cubic meter (seen in the *unit_concentration_original* column) are affected.

The corrected version of the sentence in the original publication in the fourth paragraph of section 3.3.1 is as follows:

“This trend was apparent in most media, including surface water (reaching up to 100 000 μg/L), groundwater (up to 300 μg/L), **air (up to 0.0005 μg/L)**, dust (up to 100 000 μg/kg dry weight (dw)), soil/sediment (up to 280 000 μg/kg dw), plants (up to 8000 μg/kg dw), and blood serum (up to 8000 μg/L).”

The conclusions and implications of the original publication are not affected by these errors.

## Supplementary Material





## Notes

Table S1: List of all fluoropolymer production plants (XLSX)
Extended methods and extra figures of PFAS concentrations in all media (PDF)

