# Supplementary material for: Global Inventory of Fluoropolymer Production Plants and Their Associated PFAS Environmental Contamination
Source: Environ Sci Technol. 2026 Apr 8;60(15):11688–701. doi: 10.1021/acs.est.5c18001 (PMC13098720; doi:10.1021/acs.est.5c18001)
Supplement: Supplementary file 2 [file es5c18001_si_002.pdf]

Supporting Information
for

# Global inventory of fluoropolymer production plants and their associated PFAS environmental contamination

Anna J. Miller*, Kevin Kleemann*, Juliane Glüge, Ian T. Cousins, Dorte Herzke, Rainer Lohmann, Mark F. Miller, Amanda Rensmo, Xenia Trier, Zhanyun Wang, and Martin Scheringer

*co-first authors

**Corresponding authors:**

Martin Scheringer (martin.scheringer@usys.ethz.ch)

Kevin Kleemann (kevin.kleemann@usys.ethz.ch)

## Contents:

**- Supporting Information (PDF) -** this document (24 pages)

S1. Extended details on Methods

S2. Concentrations over time, by medium and by city, for different PFAS groups and substances

S3. Concentrations over distance, by medium and by city, for different PFAS groups and substances

S4. Concentrations of all PFAS, labeled by substance, in each medium

- **SI Tables (Excel)**
  - Table S1 – list of all fluoropolymer production plants
  - Table S2 – PFAS full names, abbreviations, and their assigned group
  - Table S3 – all compiled concentration data
  - Table S4 – reference codes and full citations

## S1. Extended details on Methods

### a. Locating the fluoropolymer production plants (FPPs)

GPS coordinates for each FPP were determined using Google Maps in satellite mode along with the site names and the company-provided addresses. The coordinates provided in the supplemental information of Park et al.[1] served as a guide for the FPPs in China, which were particularly challenging to find on Google Maps due to differences in mapping systems, confidentiality, and language. Because of the difficulty in finding FPP locations in China, we classified our reported GPS coordinates (reported in SI Table S1) as “high” or “low” confidence.

“High confidence” coordinates (colored green in Table S1) indicate that a plant can be seen on Google Maps satellite view (i.e., it visually looks like an industrial manufacturing site) *and* the company name is registered at that site on Google Maps. All sites in the Unites States, continental Europe, and India, and 5 of 6 sites in Japan were determined at the high-confidence level.

“Low confidence” coordinates (colored yellow in Table S1) indicate that a visible industrial site could be found on Google Maps satellite view where the FPP could plausibly be (according to the street address), but that the company name is not specifically registered to that site. One Japanese site and 11 Chinese sites were determined at the low-confidence level.

### b. PFAS measurement data collection

For a few studies where raw data could not be obtained, we extracted data from figures in the main text or the supporting information. To extract data from figures, we used the free online software programs https://PlotDigitizer.com and https://Automeris.io. With these programs, one can upload the figure, set the x- and y-axes as a scale, and mark each data point. Then, the program determines the x and y value of each point. This method adds uncertainty and reduced precision to the data, so we indicate accordingly whether data was obtained in this way in our final data table (SI Table S3).

### c. Potentially exposed population calculations

We quantified populations within defined distances of each fluoropolymer production plant (FPP) using the Global Human Settlement Layer (GHSL) 2020 population grid (P2023A release; ~100 m resolution) within Google Earth Engine (GEE) accessed via the rgee package in R.[2] All operations were performed at the native grid scale. The population band was converted to floating-point and re-masked to replace negative or missing pixels (mainly over water) with zeros to prevent spurious values near coastlines.

Cumulative population totals were calculated as a function of distance by drawing circular buffers of 5 km to 50 km radius around each FPP in 5 km increments and summing all GHSL pixels inside each buffer. This yielded distance–population curves for each facility as well as aggregate curves for each country and five regional groupings (United States, China, Japan, Europe, and Others). The 10 km and 50 km results were extracted from these curves as examples for subsequent analyses.

To account for coordinate uncertainty, we repeated the calculation for 10 independent realizations of each FPP location in which the coordinates were randomly perturbed ("jittered") within a uniform 10 km radius of the reported position. For every facility and distance, we calculated the mean and the 10th–90th percentile range of cumulative population across these realizations, providing uncertainty bounds.

For the 10 km and 50 km distances, we also produced overlap-adjusted results to avoid double-counting in areas where buffers around nearby facilities intersect. A "share image" was created by dividing the population of each grid cell by the number of overlapping buffers, ensuring that shared populations were apportioned symmetrically among facilities. These overlap-adjusted counts were summed per facility to yield no-double-count totals. Facilities were assigned to countries using Natural Earth administrative boundaries, with nearest-boundary assignment for coastal sites, and aggregated to regional totals.

As a validation step, we cross-checked our population estimates against an independent implementation of the Global Human Settlement Layer (GHSL) population grids: Tom Forth's Population Around a Point web tool.[3] The site uses GHSL data (converted into a spatial database and queried via an API) to calculate the number of people within a user-defined radius of any point on Earth. We tested three sites (in the

UK, India, and China) at 10 km radii and confirmed that the reported populations were of the same order of magnitude as our results, consistent with expected differences due to dataset version (the website uses GHSL 2025 rather than GHSL 2020) and methodological details.

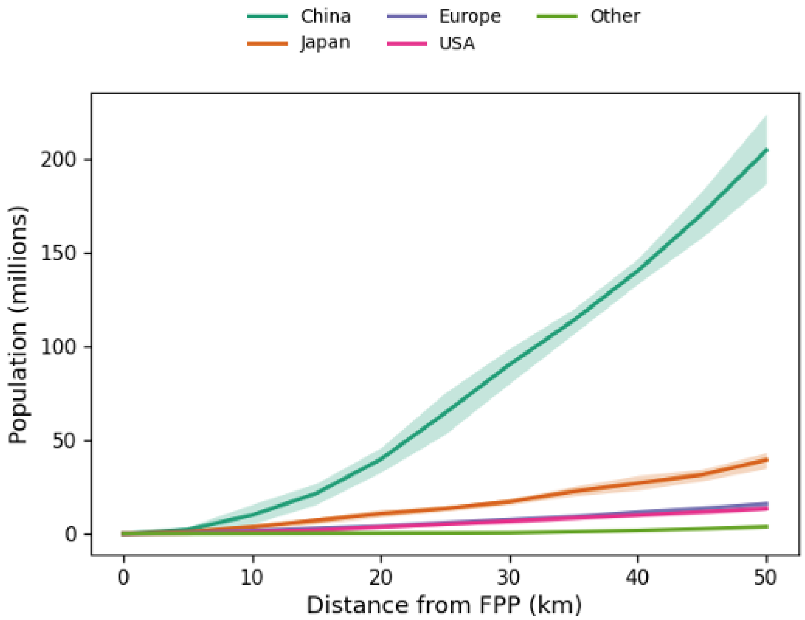


Figure S1 Estimated cumulative population living within 5–50 km of fluoropolymer production plants (FPPs), aggregated by region. Population estimates are based on the GHSL 2020 population grid at approximately 100 m resolution. Circular buffers were drawn around each FPP at 5 km increments, and populations within each buffer were summed. Results reflect the mean of 10 coordinate-jittered simulations per facility to account for location uncertainty, with shaded regions indicating the 10th–90th percentile range. Regional groupings include the United States (USA, pink), China (turquoise), Japan (orange), Europe (purple), and Others (green).

## S2. Concentrations over time

Temporal trends were assessed using measurements within 5 km of FPPs, grouped by year. $PFAS_{sum}$ concentrations measured in all media across different years are shown in **Figure S2**. **SI Figures S3–S8** show concentrations in all media for PFCAs, PFECAs, PFSAs, all other PFAS, PFOA, and HFPO-DA.

Overall, data coverage is sparse, and measurements at multiple times for the same facility and medium are rare in the peer-reviewed literature. Even so, several trends emerge from the data in **Figure S2.** First, at Pierre-Bénite (France), $PFAS_{sum}$ concentrations decreased in surface water from 2013 to 2023 and in groundwater from 2010 to 2023, while concentrations in soil and sediment have remained mostly constant from 2013 to 2024. $PFAS_{sum}$ in groundwater also shows decreases for the FPP in Osaka from 2005 to 2015 and in Fayetteville from 2018 to 2023. Decreases likely reflect stricter emission controls, although concentrations have still remained high on the global scale. In surface water at the Huantai County and Spinetta FPPs, while there are measurements across years, there is no apparent significant increase or decrease in $PFAS_{sum}$. At the remaining sites, there is not enough data to indicate any site-specific temporal trends in $PFAS_{sum}$, although it is evident that across all sites, $PFAS_{sum}$ remains largely constant over the years.

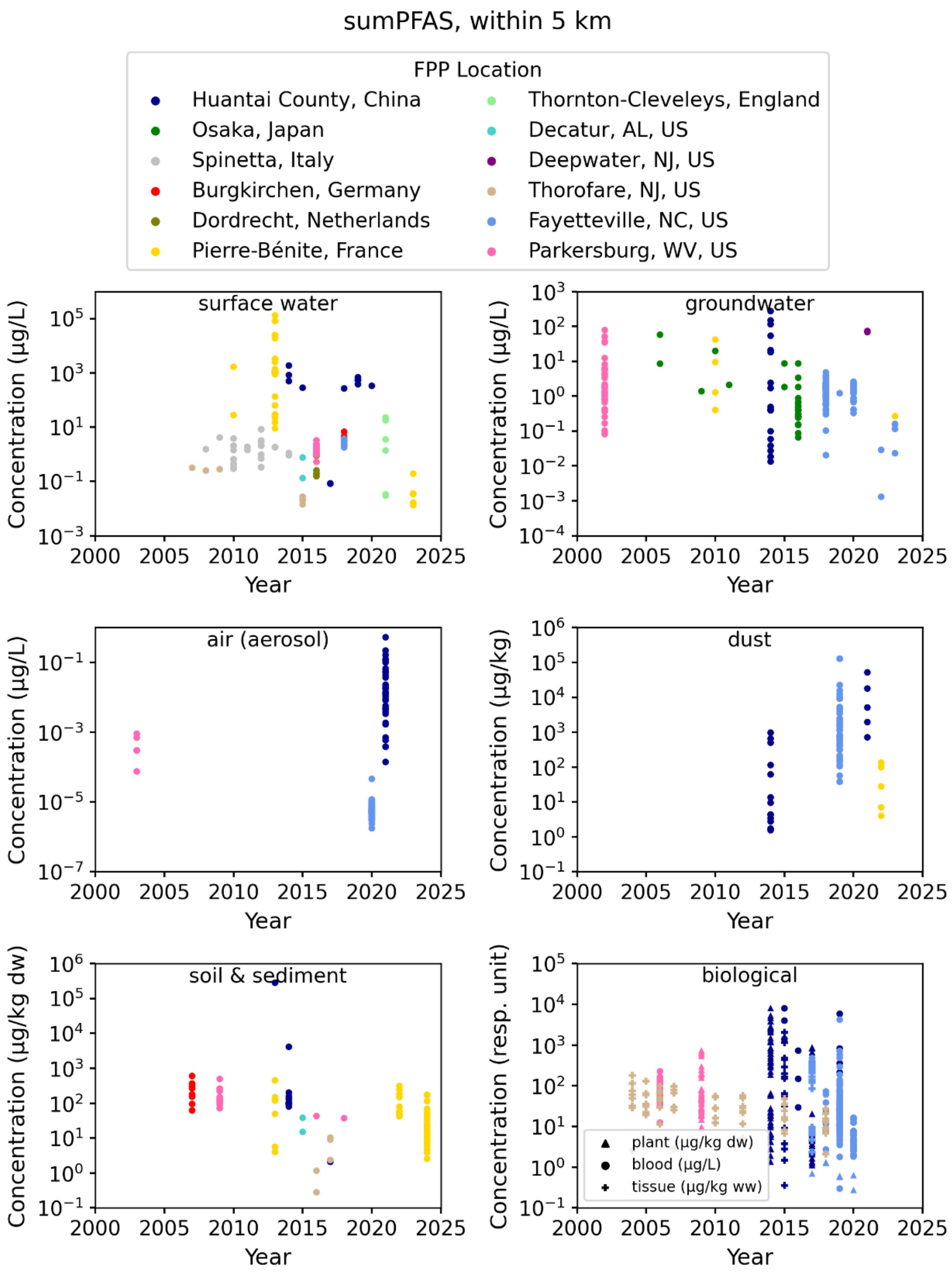


Figure S2. PFAS$_{sum}$ concentrations measured within 5 km of a fluoropolymer production plant (FPP), according to the year it was measured and the medium it was measured in. Colors refer to the location of the FPP. Only downstream measurements are plotted for surface water and sediment. Only dry weight (dw) concentrations are plotted for soil and sediment. All measurements have been previously reported; see **Table S3** for the complete table of data and sources.

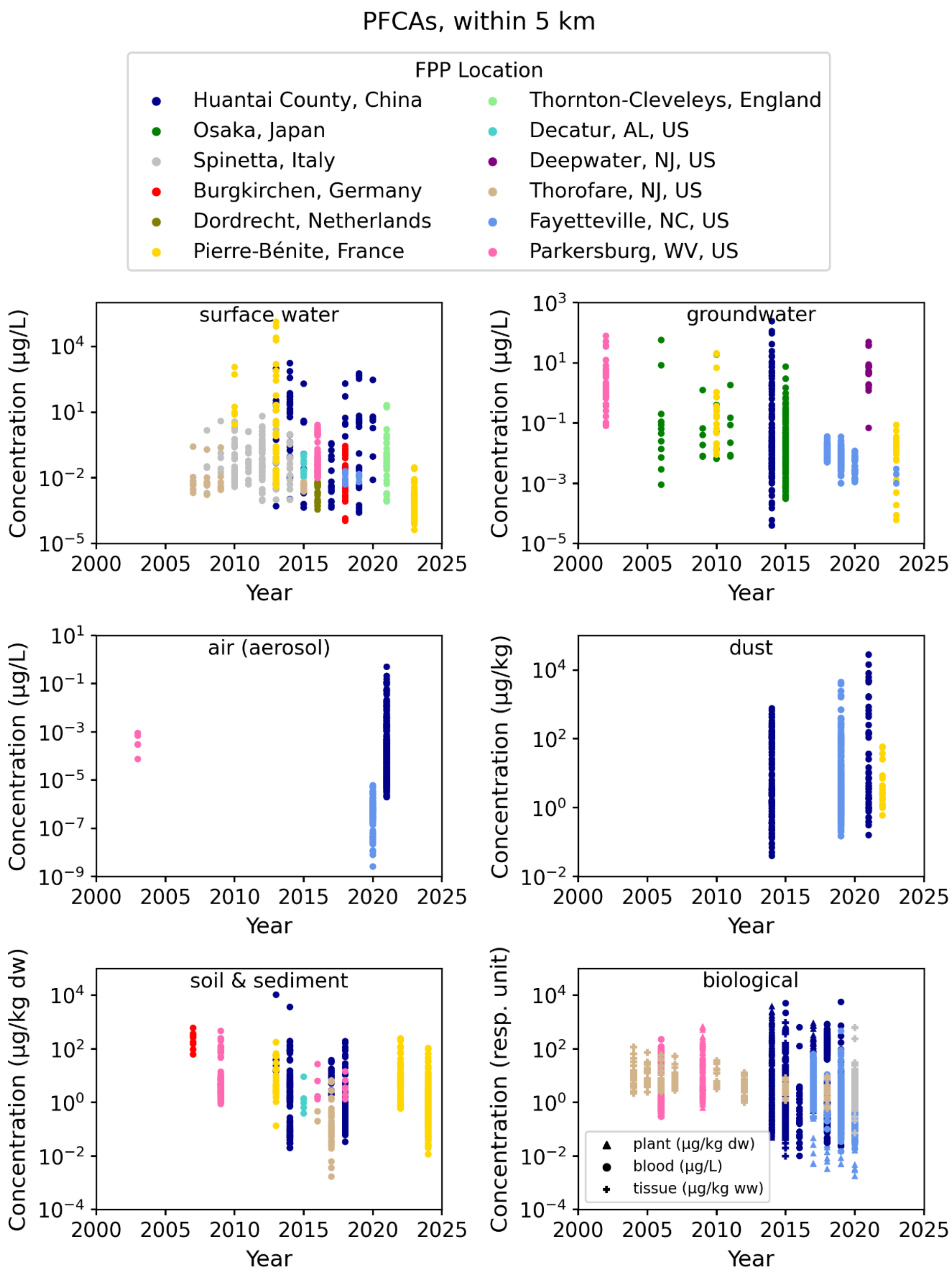


Figure S3. Concentrations of all individual **PFCA** substances measured within 5 km of the fluoropolymer production plant, with the year it was measured in each medium (surface water, groundwater, air, dust, soil/sediment, plants, and animals), colored by facility location.

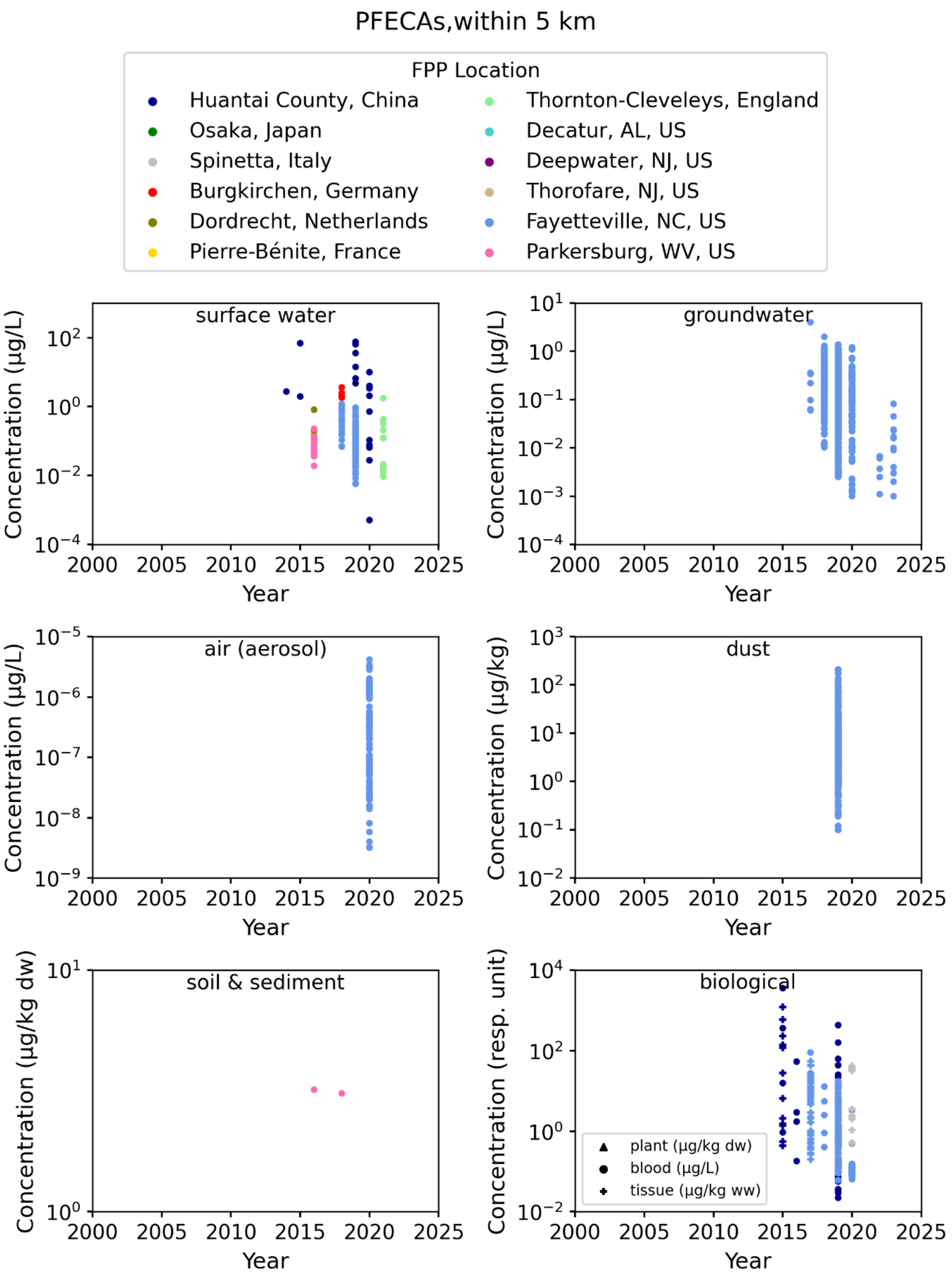


Figure S4. Concentrations of all individual **PFECA** substances measured within 5 km of the fluoropolymer production plant, with the year it was measured in each medium (surface water, groundwater, air, dust, soil/sediment, plants, and animals), colored by facility location.

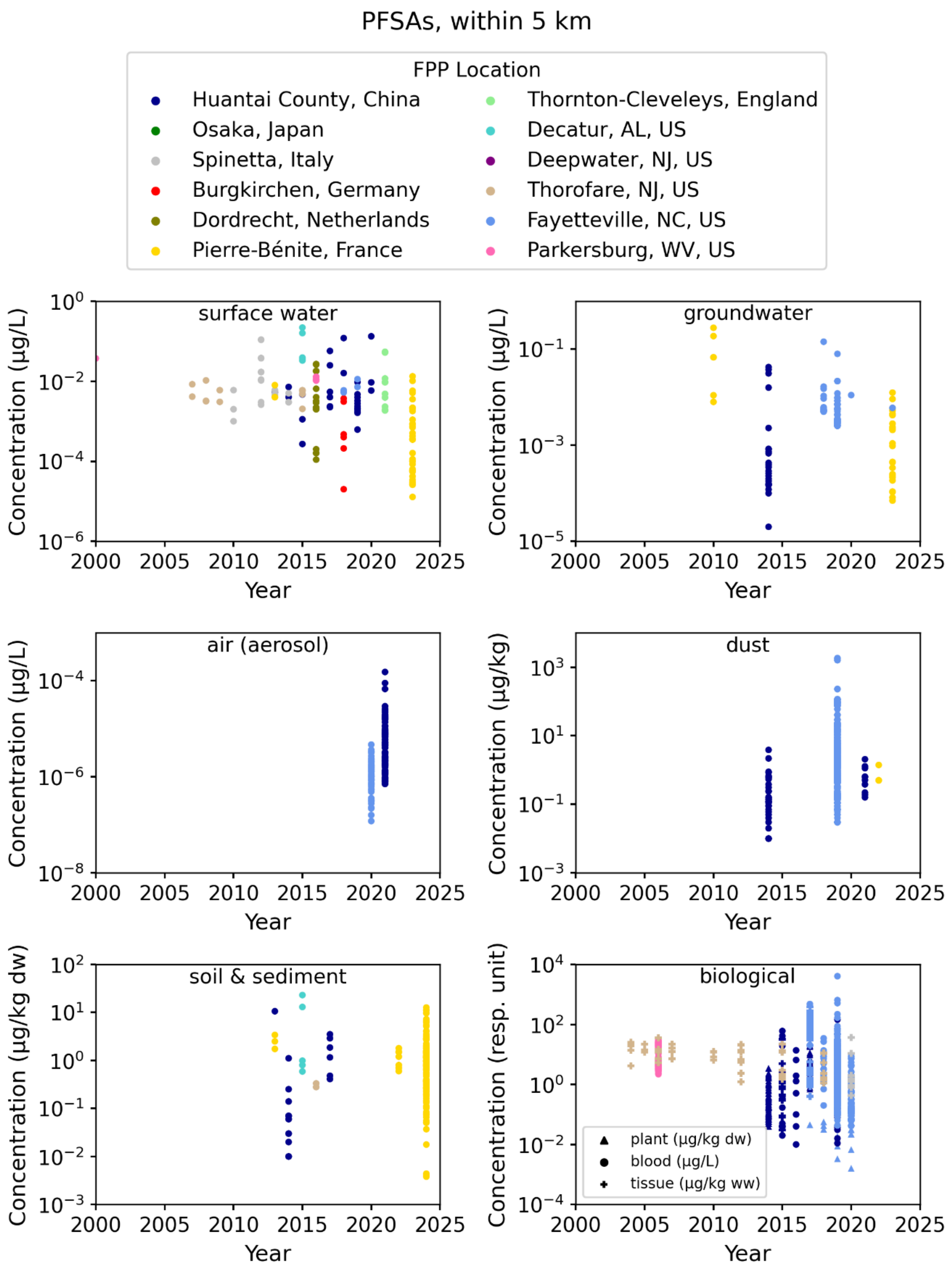


Figure S5. Concentrations of all individual **PFSA** substances measured within 5 km of the fluoropolymer production plant, with the year it was measured in each medium (surface water, groundwater, air, dust, soil/sediment, plants, and animals), colored by facility location.

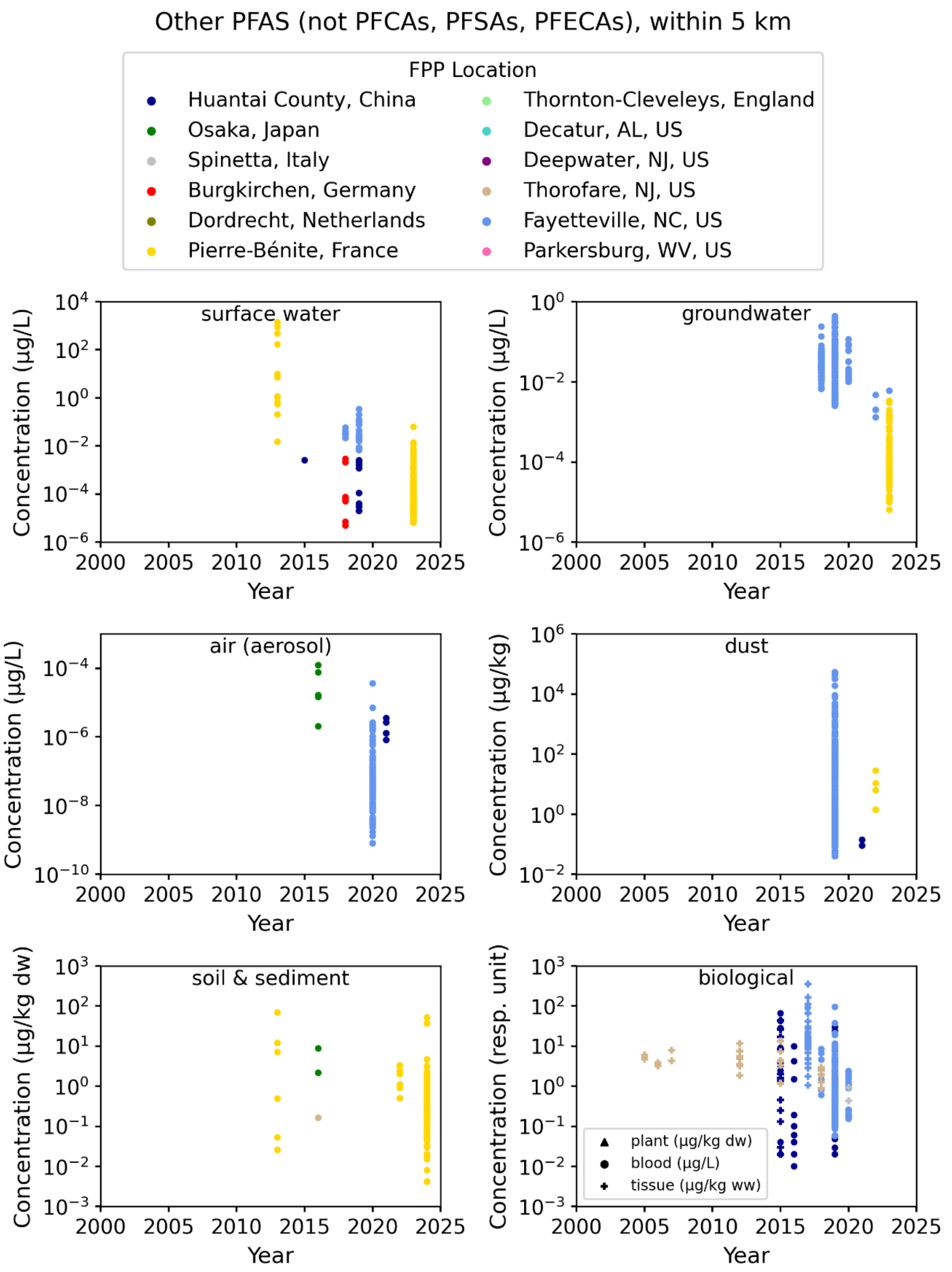


Figure S6. Concentrations of **other PFAS** substances (those which are not PFCAs, PFSAs, or PFECAs) measured within 5 km of the fluoropolymer production plant, with the year it was measured in each medium (surface water, groundwater, air, dust, soil/sediment, plants, and animals), colored by facility location.

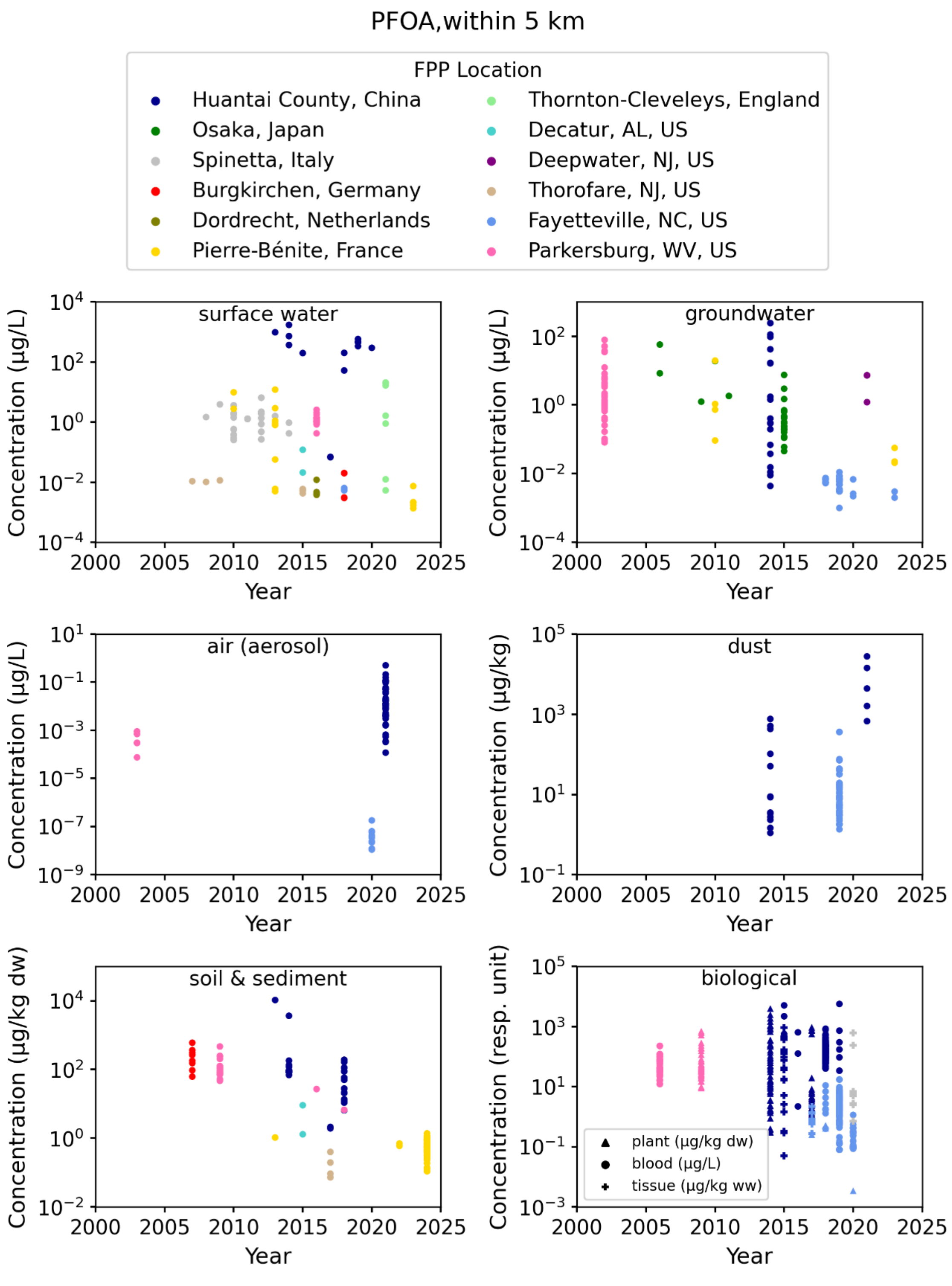


Figure S7. Concentrations of **PFOA** measured within 5 km of the fluoropolymer production plant, with the year it was measured in each medium (surface water, groundwater, air, dust, soil/sediment, plants, and animals), colored by facility location.

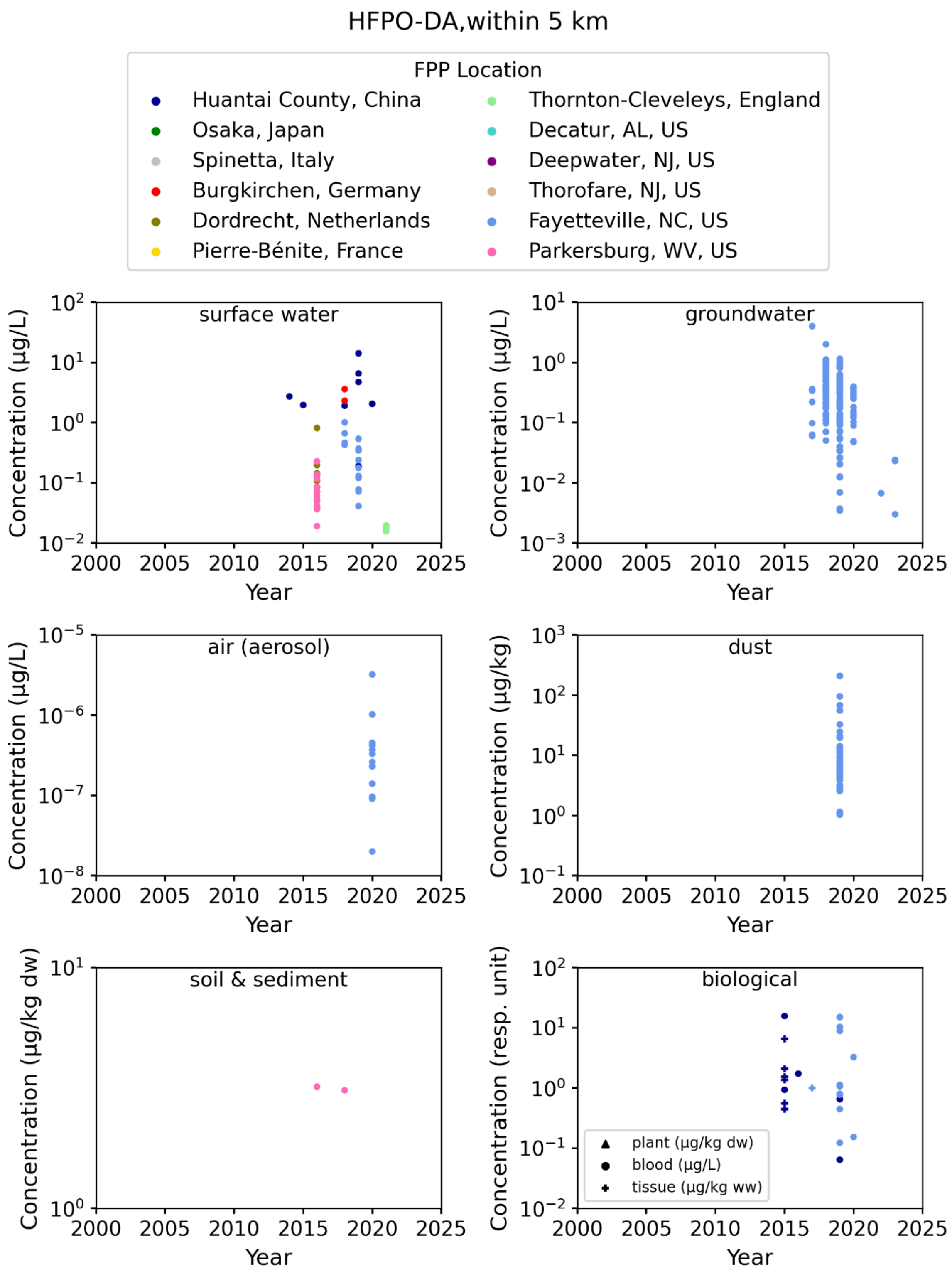


Figure S8. Concentrations of **HFPO-DA** measured within 5 km of the fluoropolymer production plant, with the year it was measured in each medium (surface water, groundwater, air, dust, soil/sediment, plants, and animals), colored by facility location.

## S3. Concentrations over distance from site

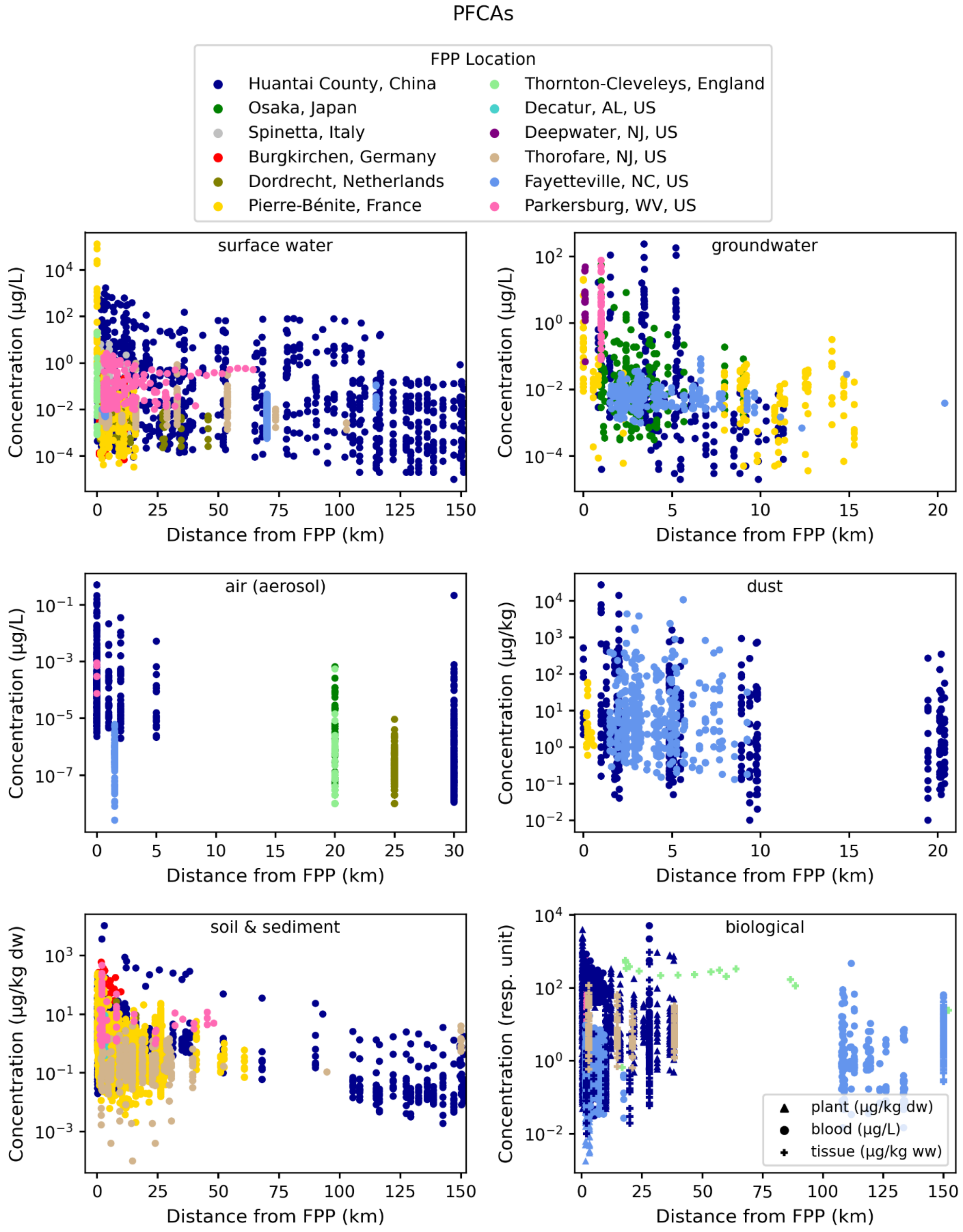


Figure S9. Concentrations of all individual **PFCA** substances at varying distances (km) from the fluoropolymer production plant (FPP) in each medium.

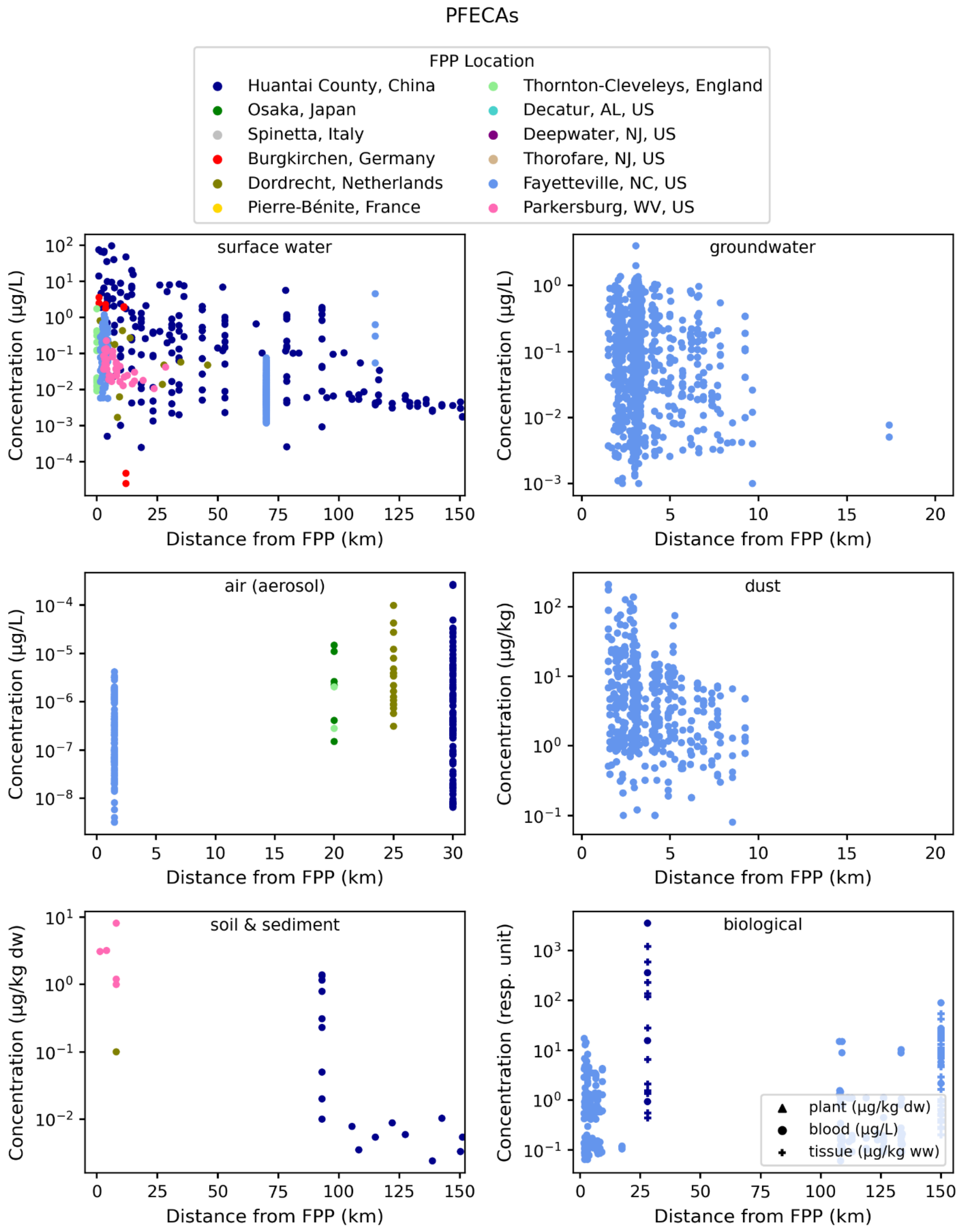


Figure S10. Concentrations of all individual **PFECA** substances at varying distances (km) from the fluoropolymer production plant (FPP) in each medium.

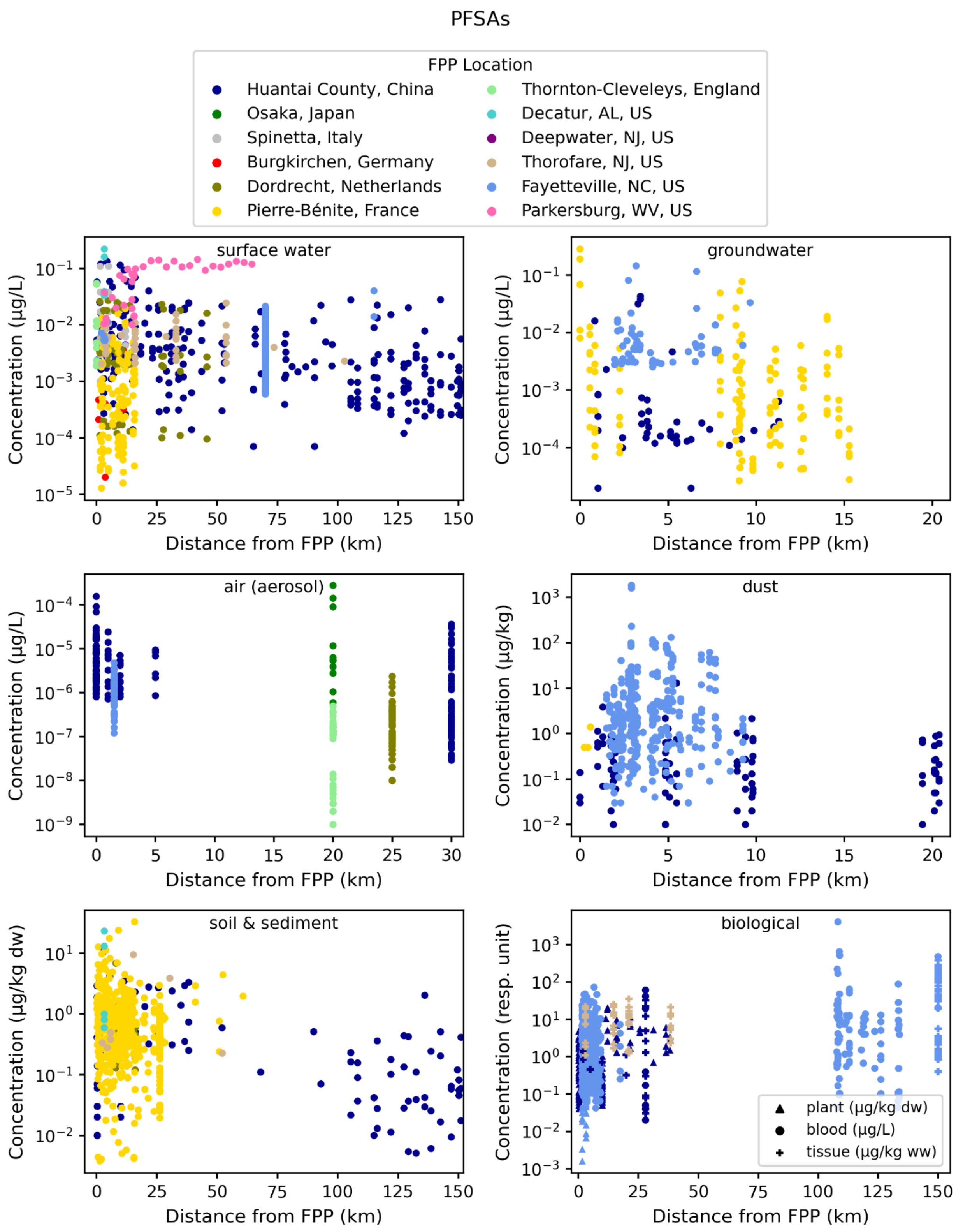


Figure S11. Concentrations of all individual **PFSA** substances at varying distances (km) from the fluoropolymer production plant (FPP) in each medium.

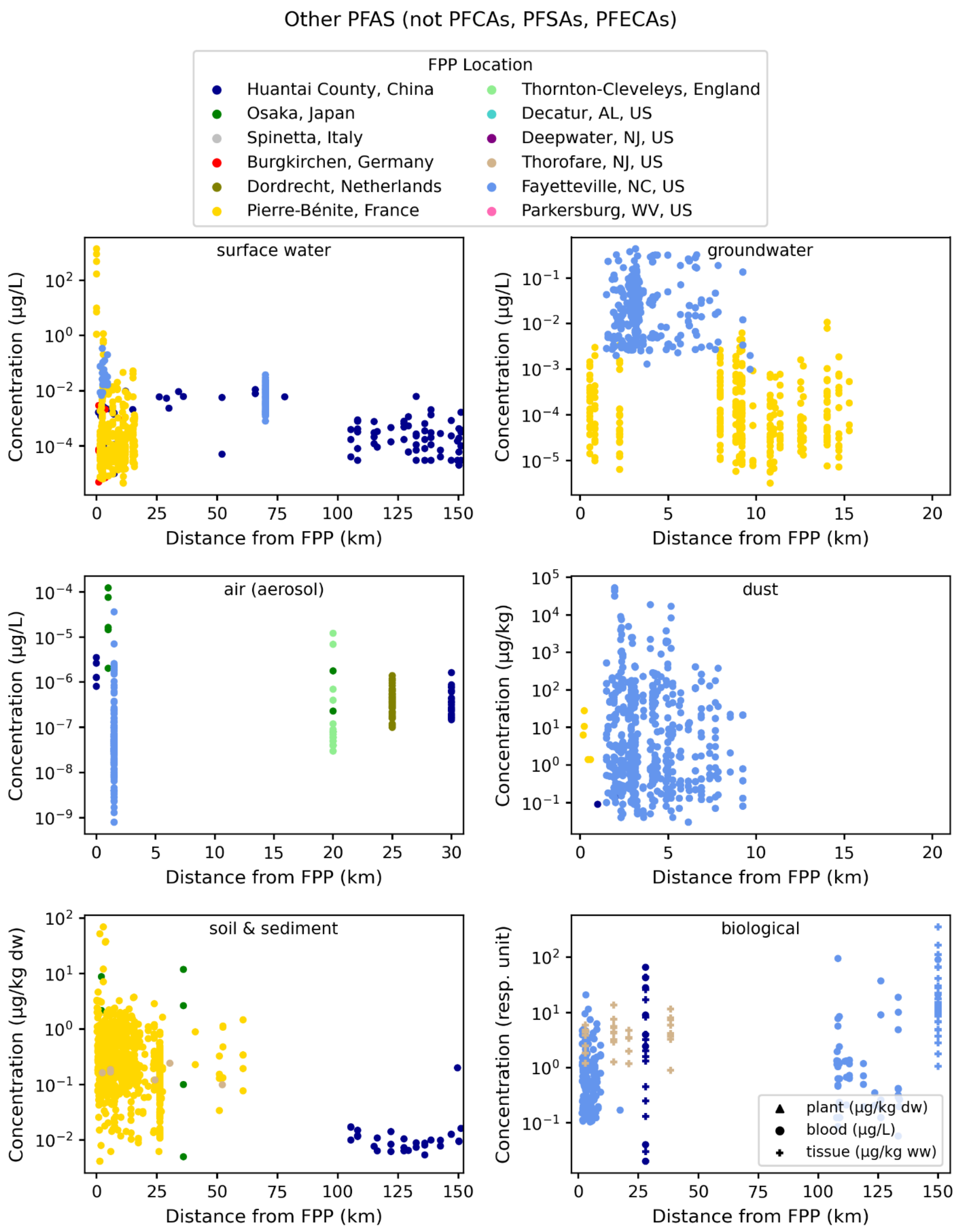


Figure S12. Concentrations of all **other PFAS** substances (those which are not PFCAs, PFSAs, or PFECAs) at varying distances (km) from the fluoropolymer production plant (FPP) in each medium.

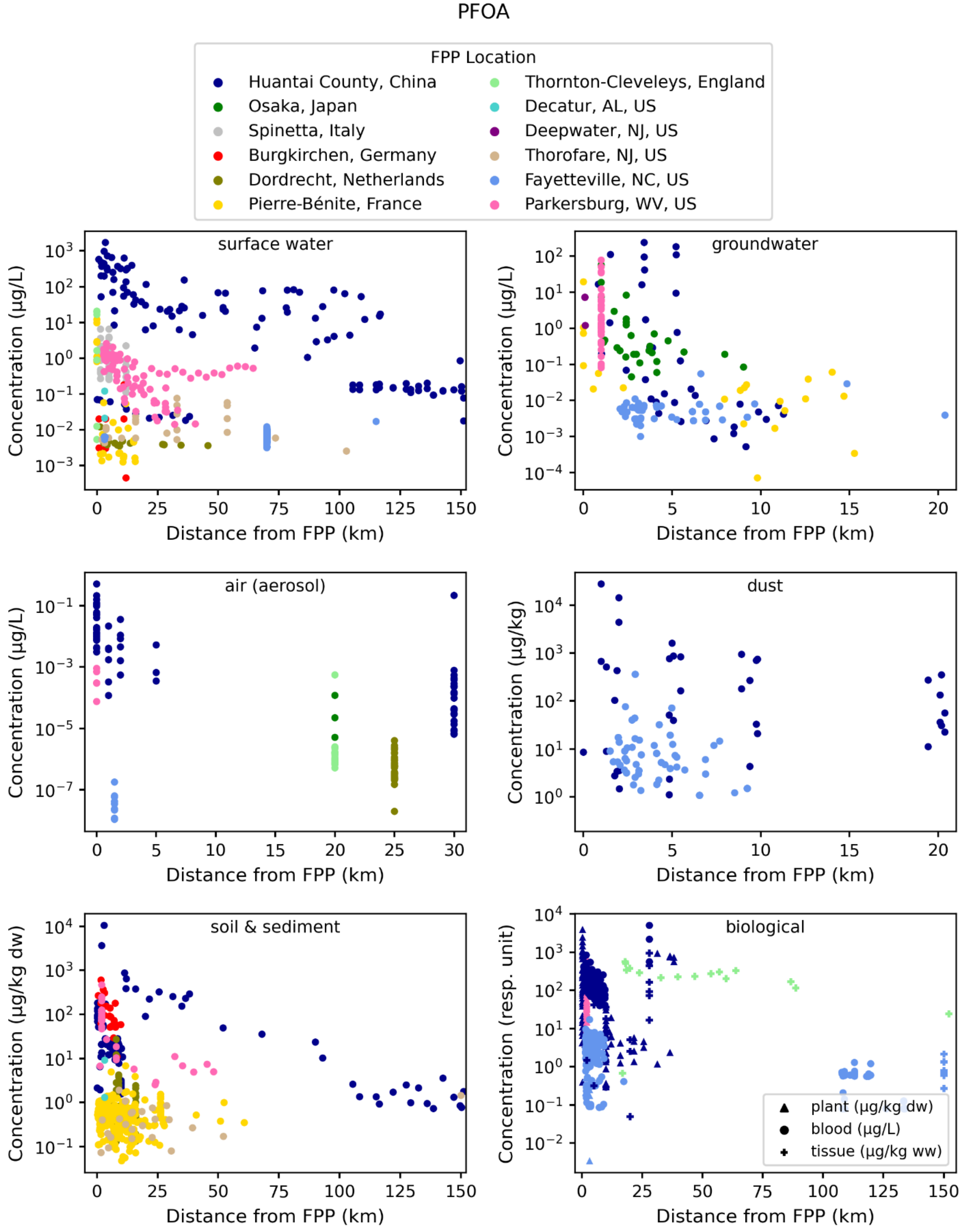


Figure S13. Concentrations of **PFOA** at varying distances (km) from the fluoropolymer production plant (FPP) in each medium.

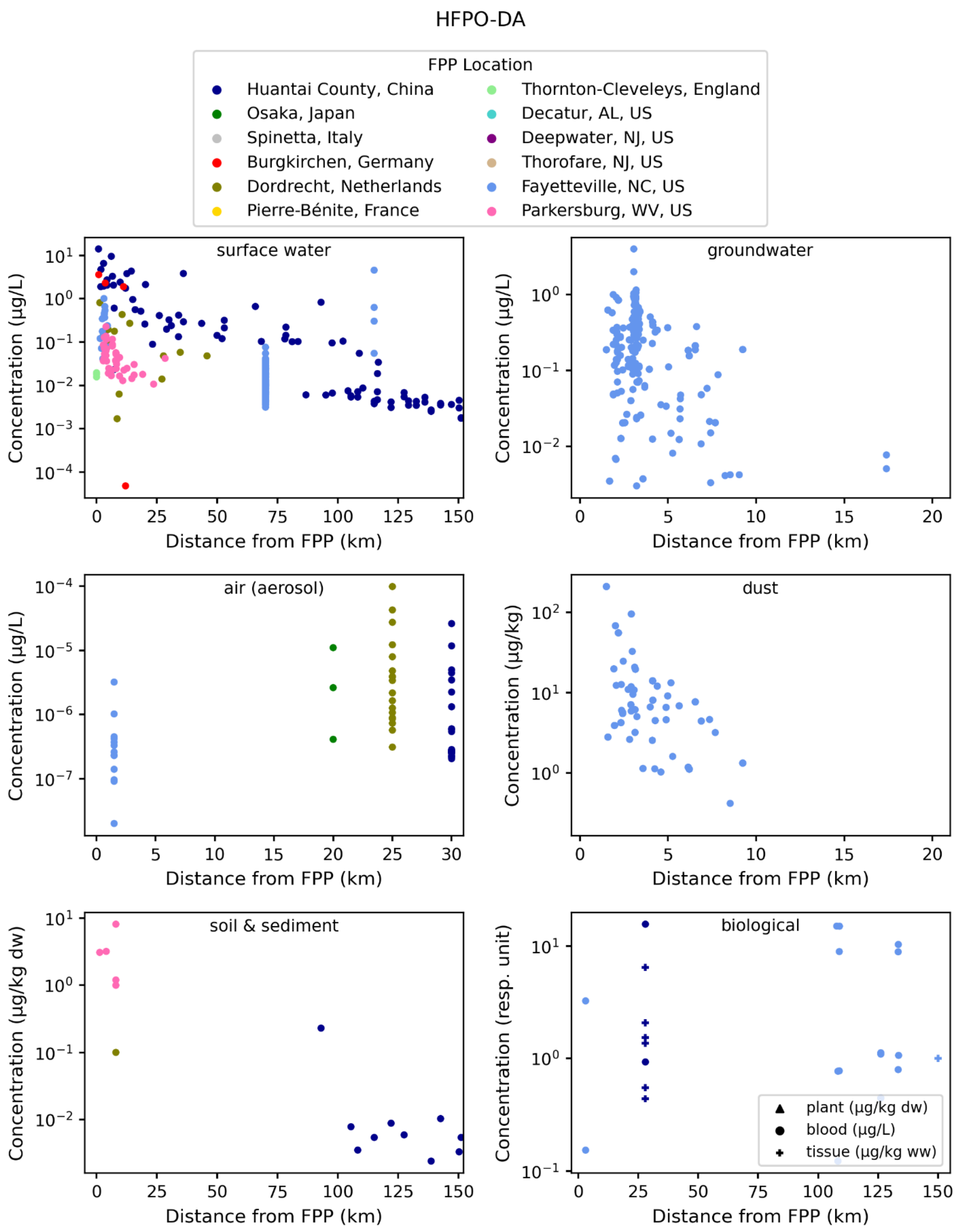


Figure S14. Concentrations of **HFPO-DA** at varying distances (km) from the fluoropolymer production plant (FPP) in each medium.

## S4. Concentrations of each PFAS substance

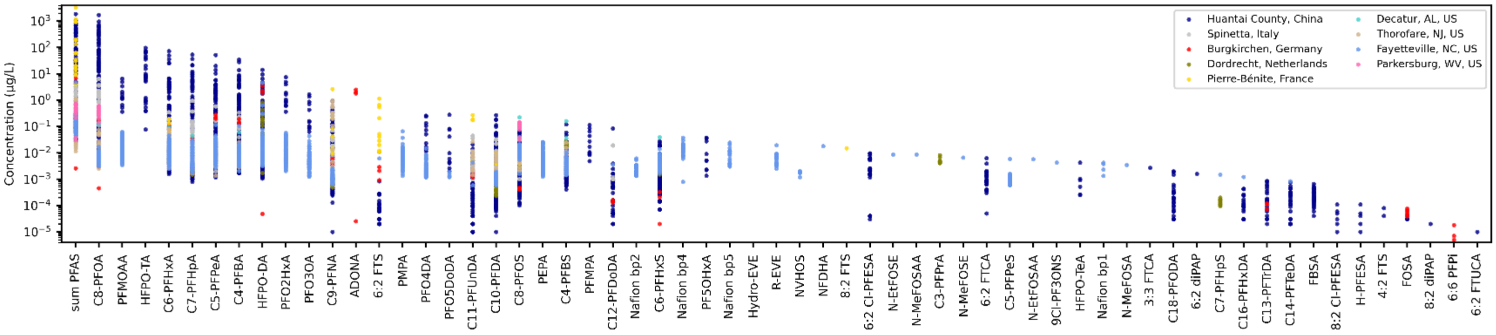


Figure S15. Concentrations of all PFAS substances detected in **surface water** near a fluoropolymer production plant.

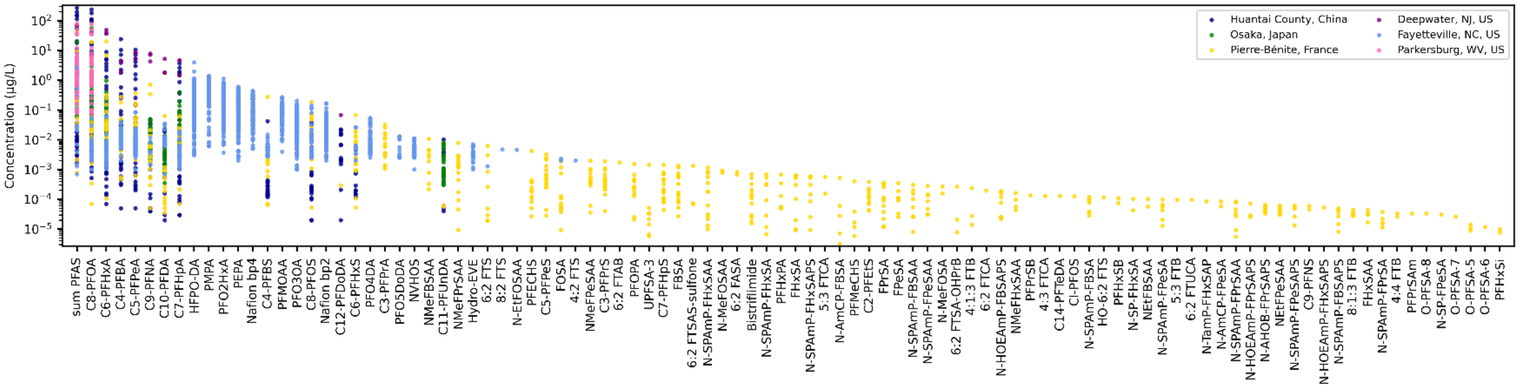


Figure S16. Concentrations of all PFAS substances detected in **groundwater** near a fluoropolymer production plant.

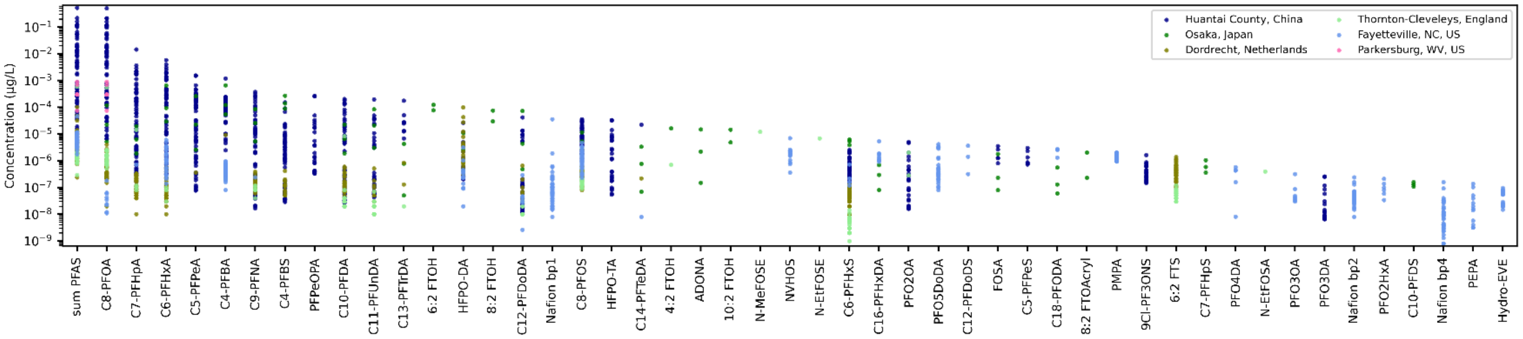


Figure S17. Concentrations of all PFAS substances detected in **air** near a fluoropolymer production plant.

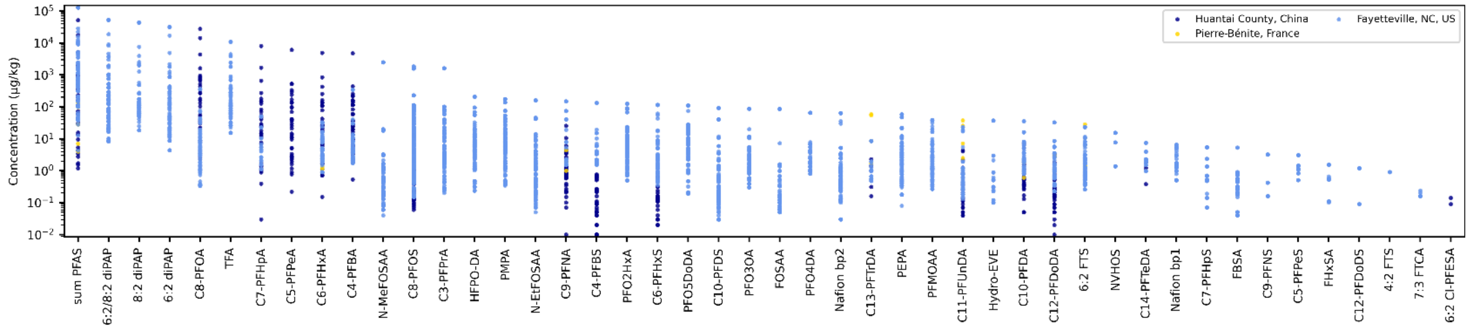


Figure S18. Concentrations of all PFAS substances detected in **dust** near a fluoropolymer production plant.

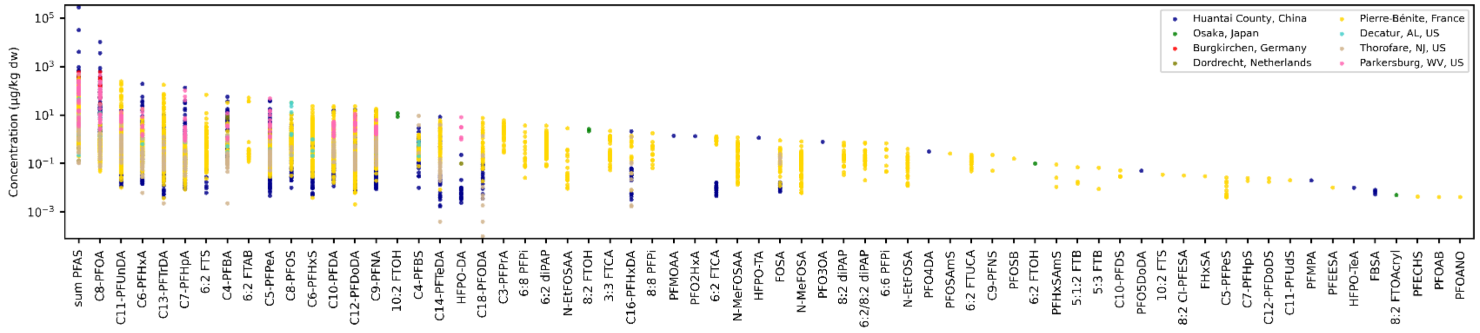


Figure S19. Concentrations of all PFAS substances detected in **soil and sediment** near a fluoropolymer production plant.

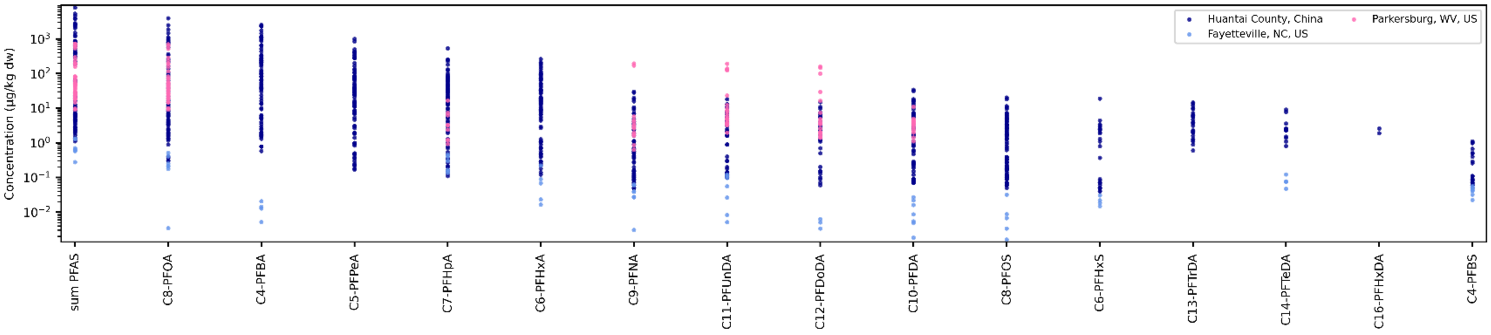


Figure S20. Concentrations of all PFAS substances detected in **plants** near a fluoropolymer production plant.

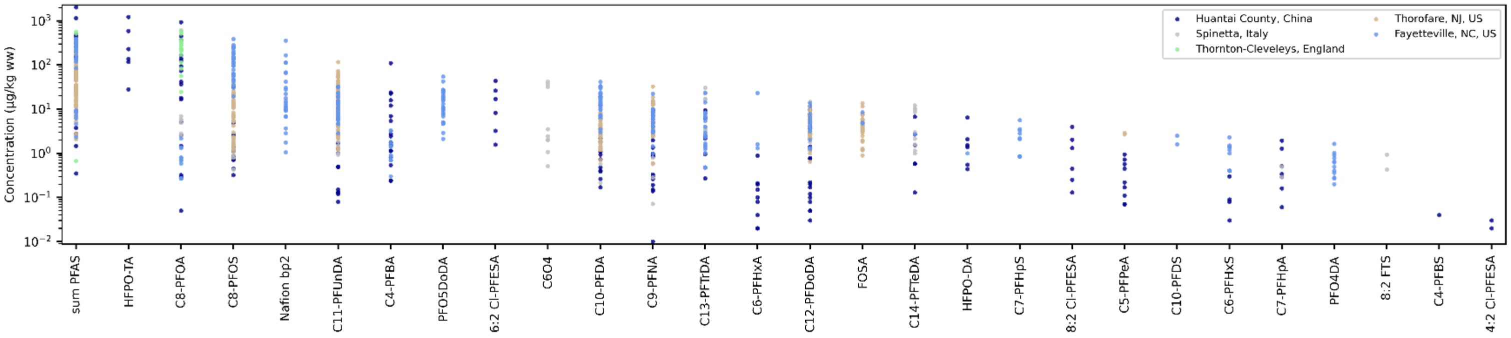


Figure S21. Concentrations of all PFAS substances detected in **animal tissue** near a fluoropolymer production plant.

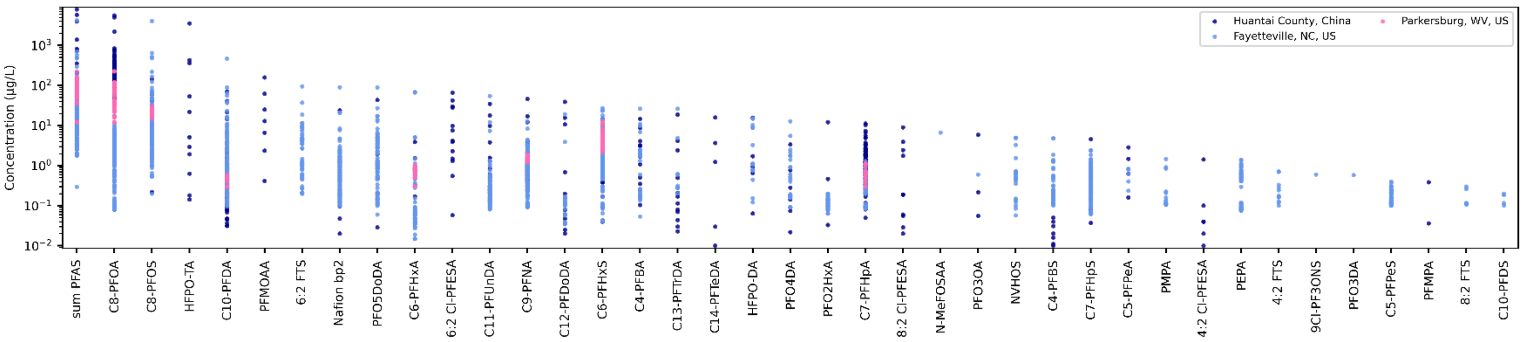


Figure 22. Concentrations of all PFAS substances detected in **animal or human blood serum** near a fluoropolymer production plant.

## References


(1) Park, H.; Kim, J.; Choi, H.; Geum, S.; Kim, Y.; Thompson, R. L.; Mühle, J.; Salameh, P. K.; Harth, C. M.; Stanley, K. M.; O'Doherty, S.; Fraser, P. J.; Simmonds, P. G.; Krummel, P. B.; Weiss, R. F.; Prinn, R. G.; Park, S. A Rise in HFC-23 Emissions from Eastern Asia since 2015. *Atmospheric Chem. Phys.* **2023**, *23* (16), 9401–9411. https://doi.org/10.5194/acp-23-9401-2023.

(2) Pesaresi, M.; Florczyk, A.; Schiavina, M.; Melchiorri, M.; Maffenini, L. GHS-SMOD R2019A - GHS Settlement Layers, Updated and Refined REGIO Model 2014 in Application to GHS-BUILT R2018A and GHS-POP R2019A, Multitemporal (1975-1990-2000-2015) - OBSOLETE RELEASE, 2019. https://doi.org/10.2905/42E8BE89-54FF-464E-BE7B-BF9E64DA5218.

(3) Forth, T. *Population around a point*. https://www.tomforth.co.uk/circlepopulations/ (accessed 2025-09-25).
