# Supplementary material for: Correction to “Global Inventory of Fluoropolymer Production Plants and Their Associated PFAS Environmental Contamination”
Source: Environ Sci Technol. 2026 Aug 29;60(36):25947–8. doi: 10.1021/acs.est.6c12429 (PMC13585140; doi:10.1021/acs.est.6c12429)
Supplement: Supplementary file 1 [file es6c12429_si_002.pdf]

Supplementary Information to
Correction to
"Global inventory of fluoropolymer production plants and their associated PFAS environmental contamination"

Anna J. Miller, Kevin Kleemann, Juliane Glüge, Rainer Lohmann, Ian T. Cousins, Dorte Herzke, Mark F. Miller, Amanda Rensmo, Xenia Trier, Zhanyun Wang, and Martin Scheringer

Original publication:
Anna J. Miller, Kevin Kleemann, Juliane Glüge, Rainer Lohmann, Ian T. Cousins, Dorte Herzke, Mark F. Miller, Amanda Rensmo, Xenia Trier, Zhanyun Wang, Martin Scheringer; Global Inventory of Fluoropolymer Production Plants and Their Associated PFAS Environmental Contamination. *Environ. Sci. Technol.* 21 April 2026; 60 (15): 11688–11701.
https://doi.org/10.1021/acs.est.5c18001

Contents:

- Figures S1 – S13 here are the corrected versions of the Figs. S2 – S14 of the original publication. Figure S14 is the corrected version of the original Fig. S17.
- SI Table S1 (Excel file) is the corrected version of SI Table S3 of the original publication.

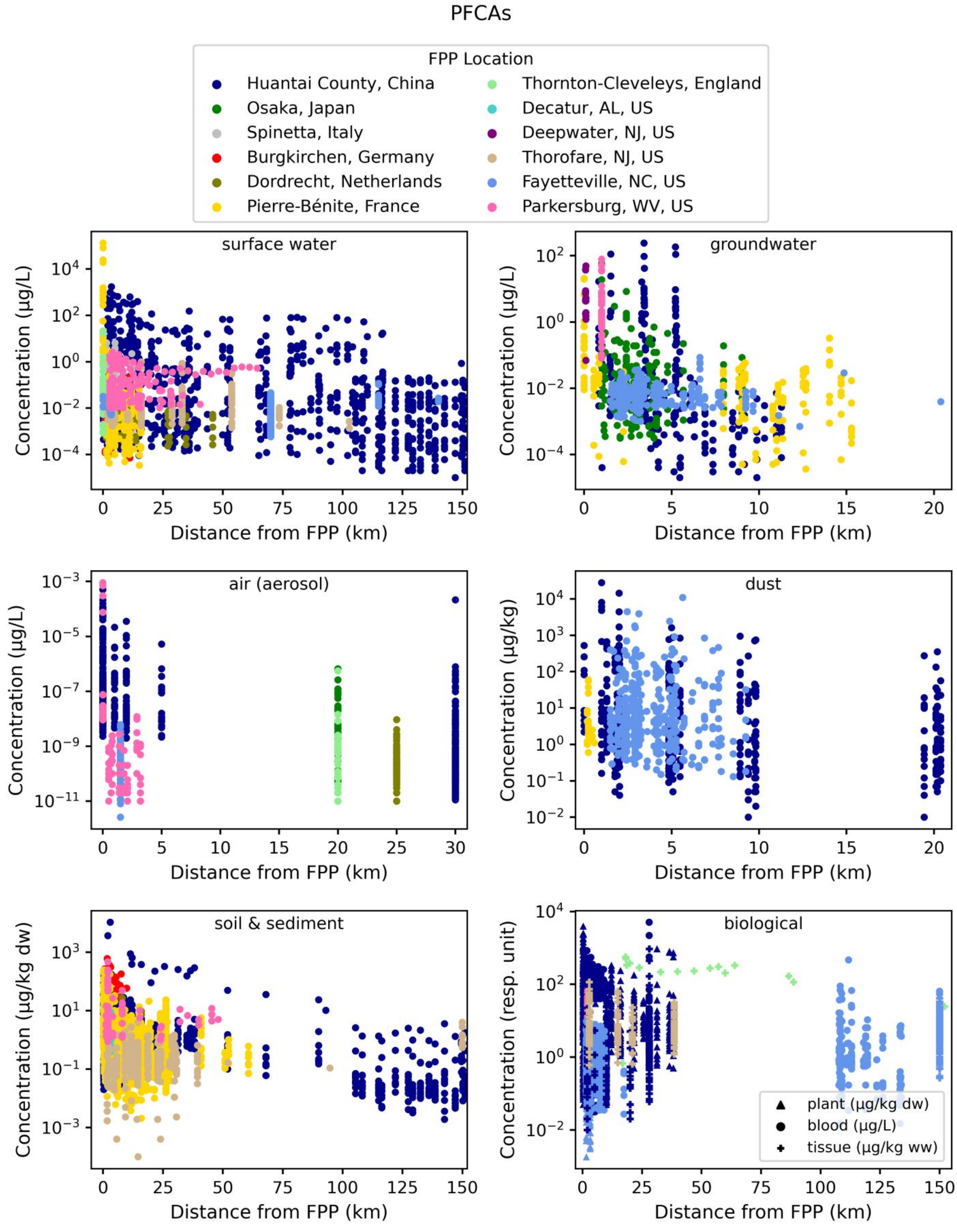

Figure S1. Corrected version of original Figure S2.

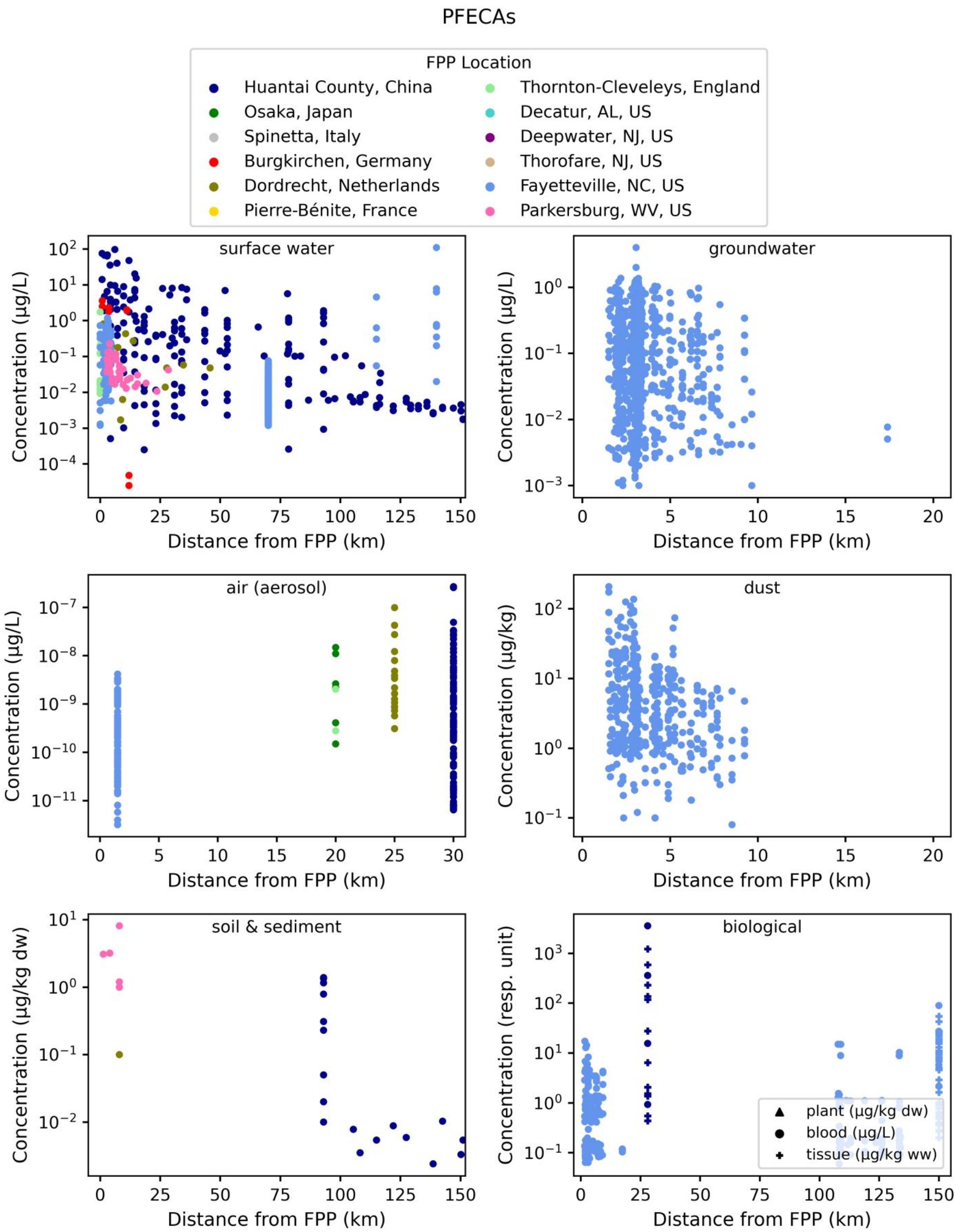

Figure S2. Corrected version of original Figure S3.

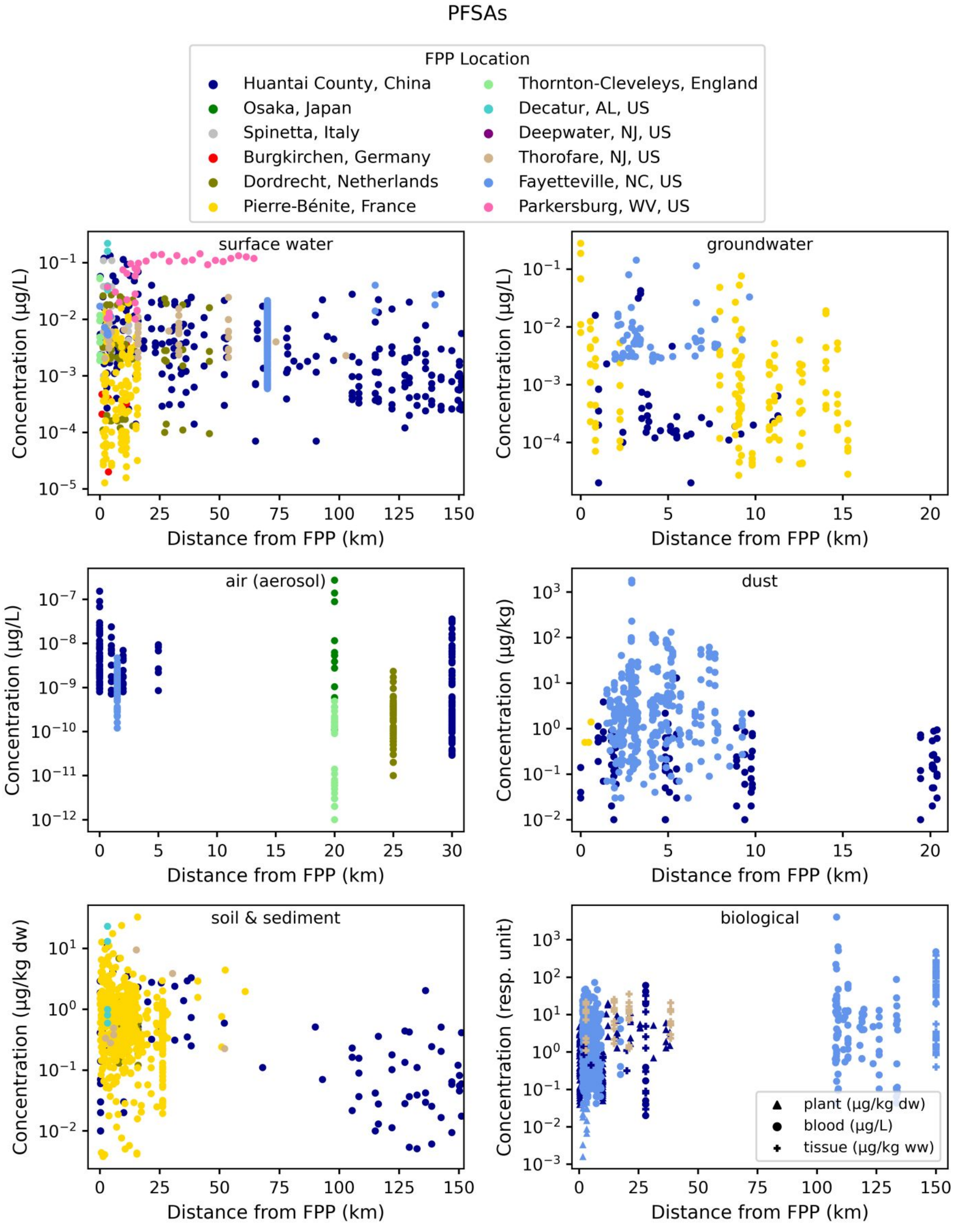

Figure S3. Corrected version of original Figure S4.

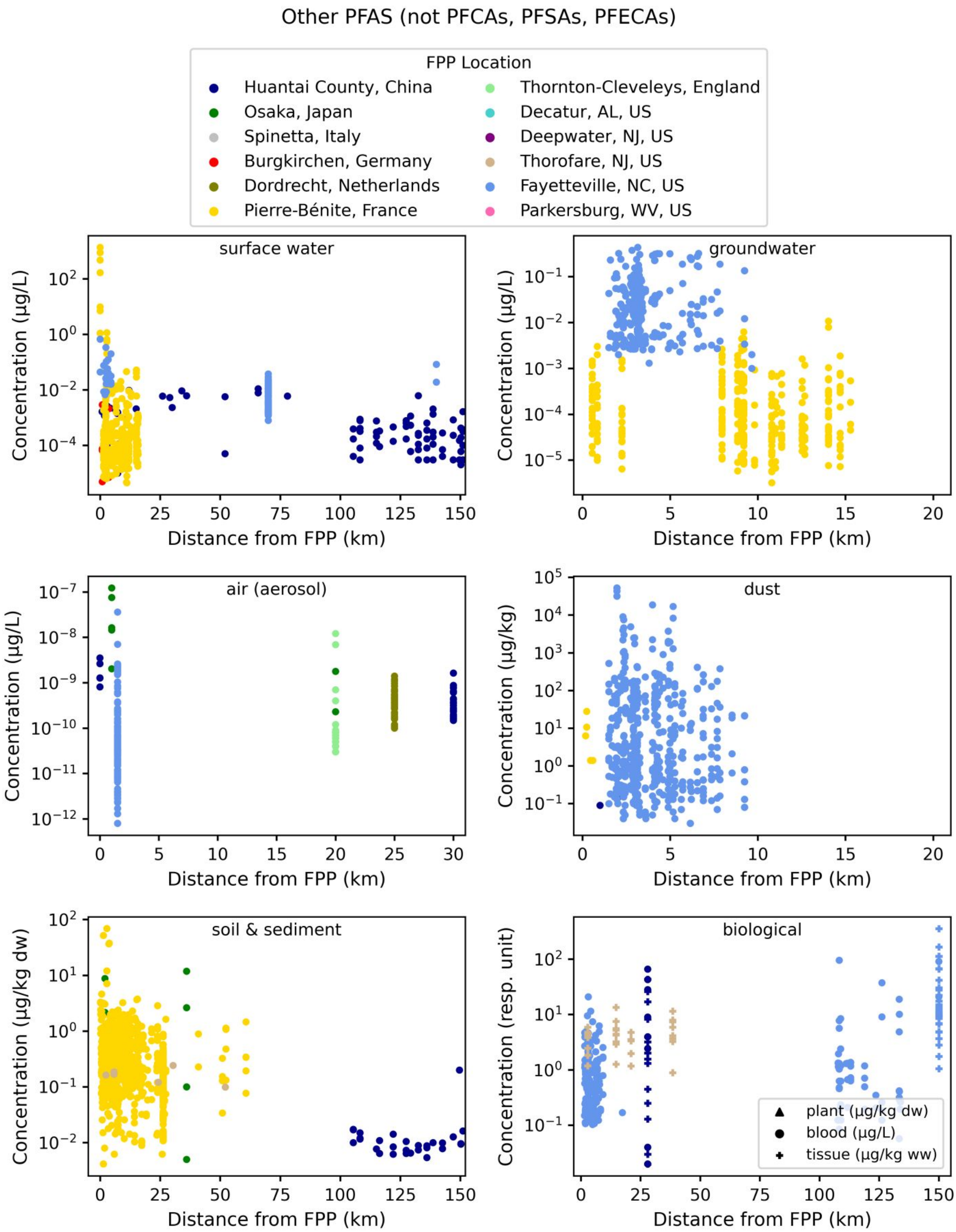

Figure S4. Corrected version of original Figure S5.

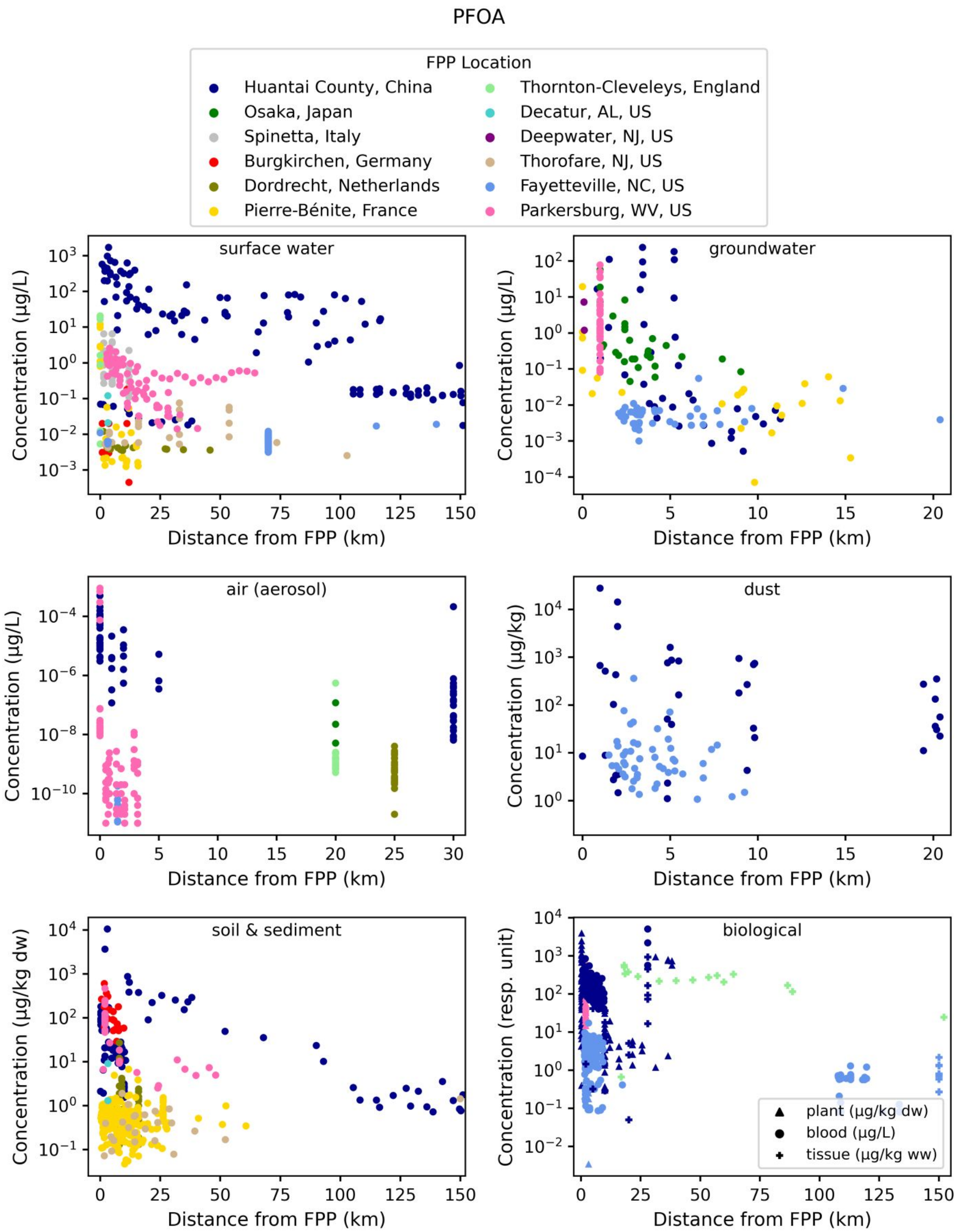

Figure S5. Corrected version of original Figure S6.

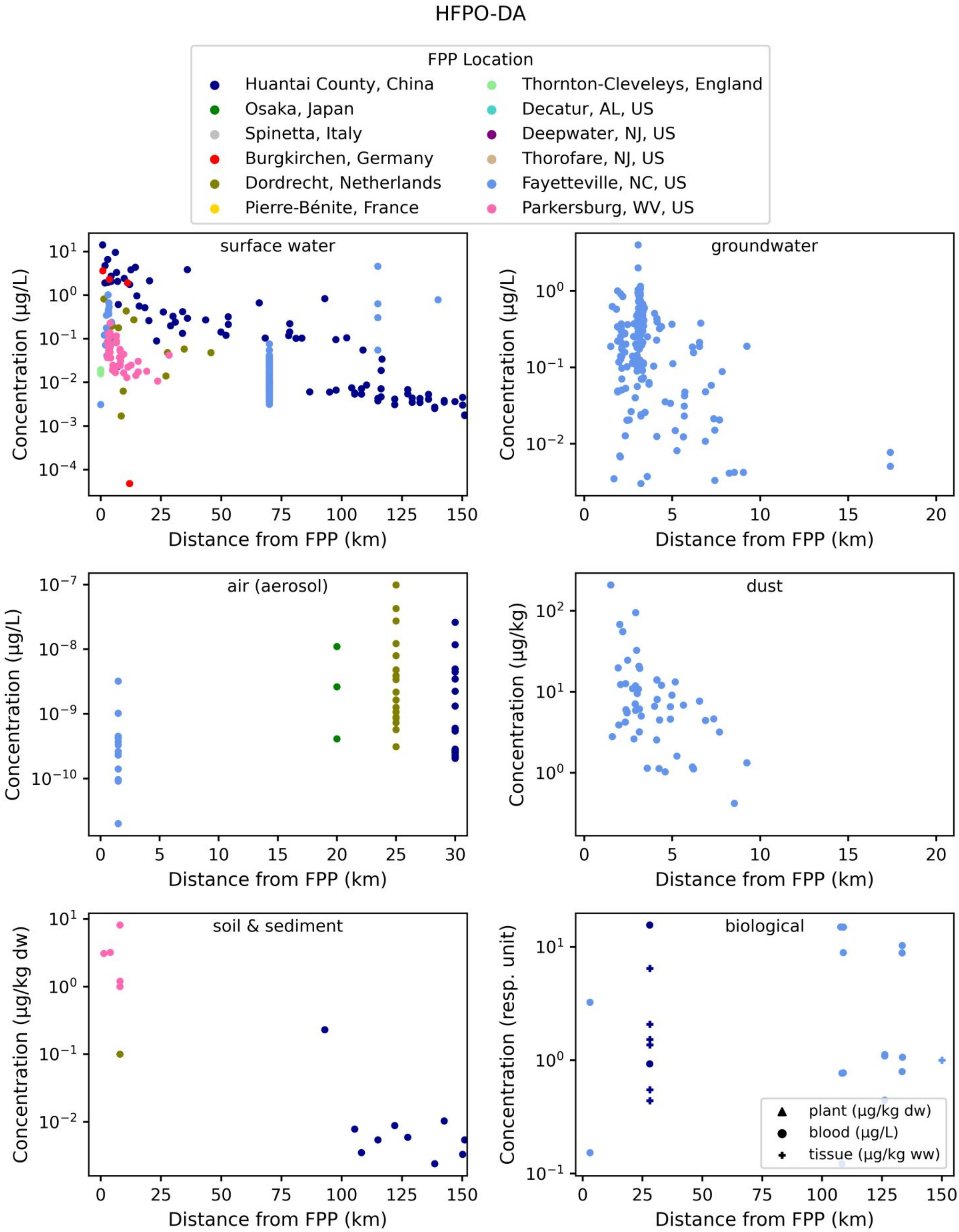

Figure S6. Corrected version of original Figure S7.

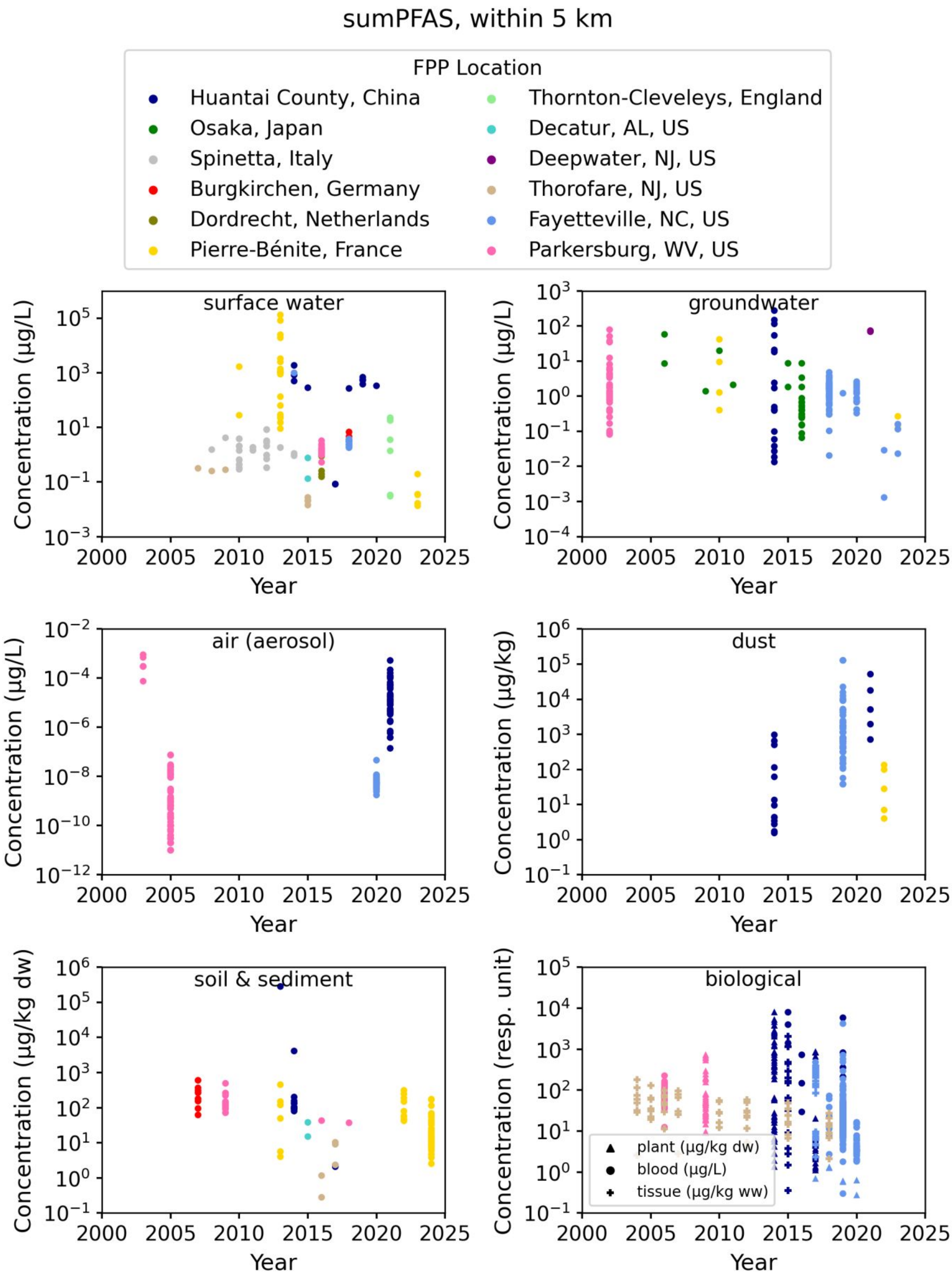

Figure S7. Corrected version of original Figure S8.

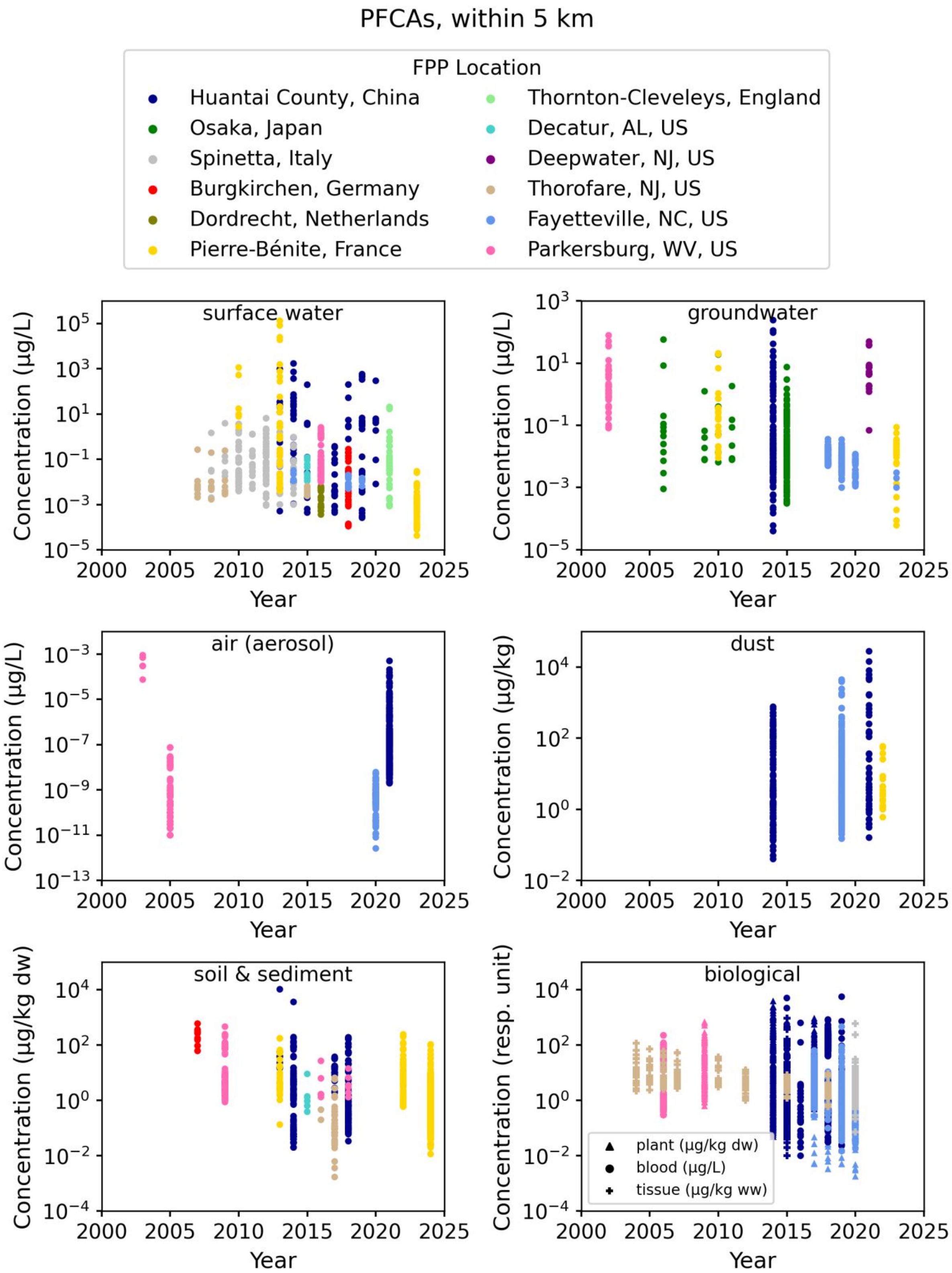

Figure S8. Corrected version of original Figure S9.

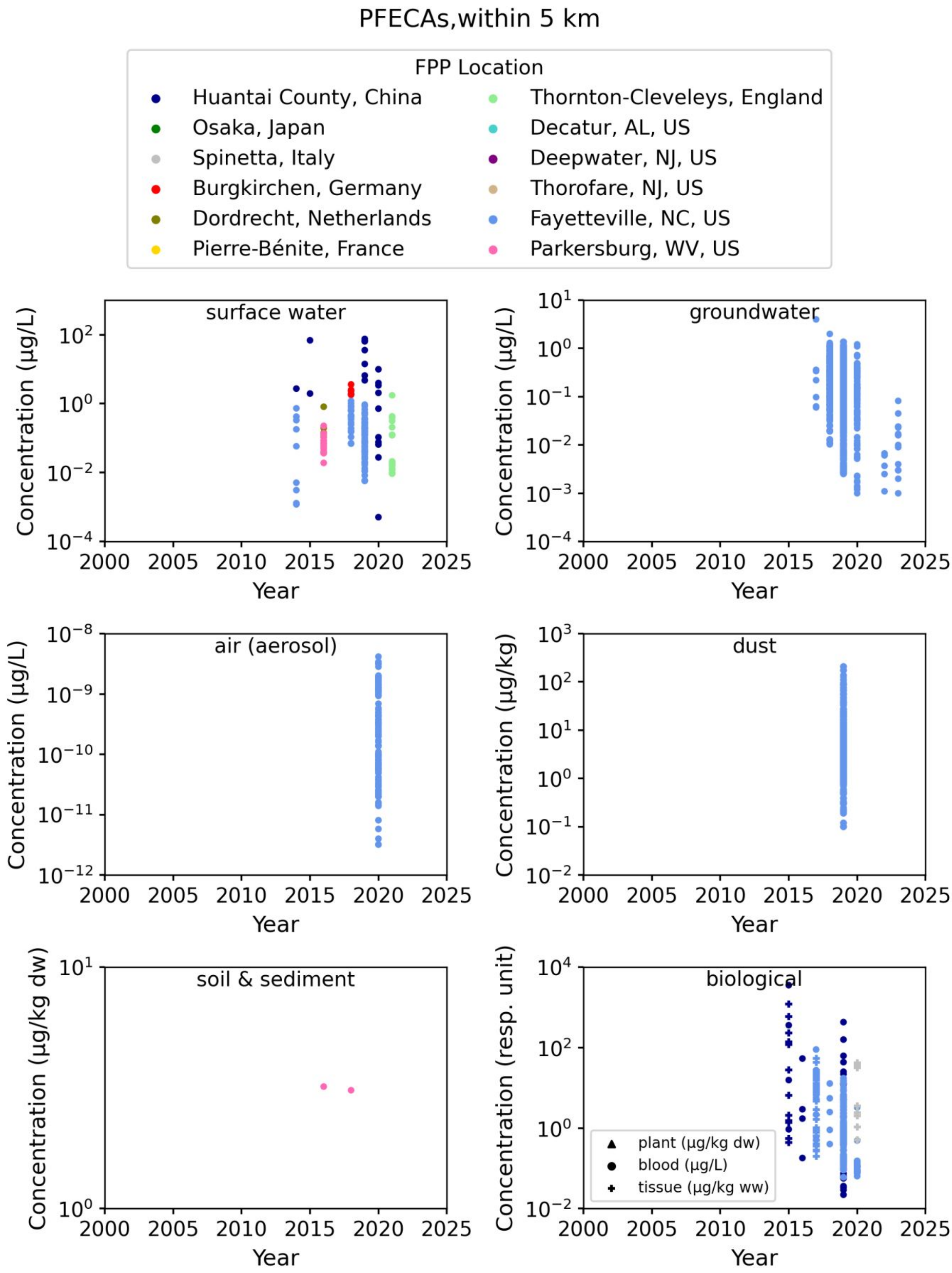

Figure S9. Corrected version of original Figure S10.

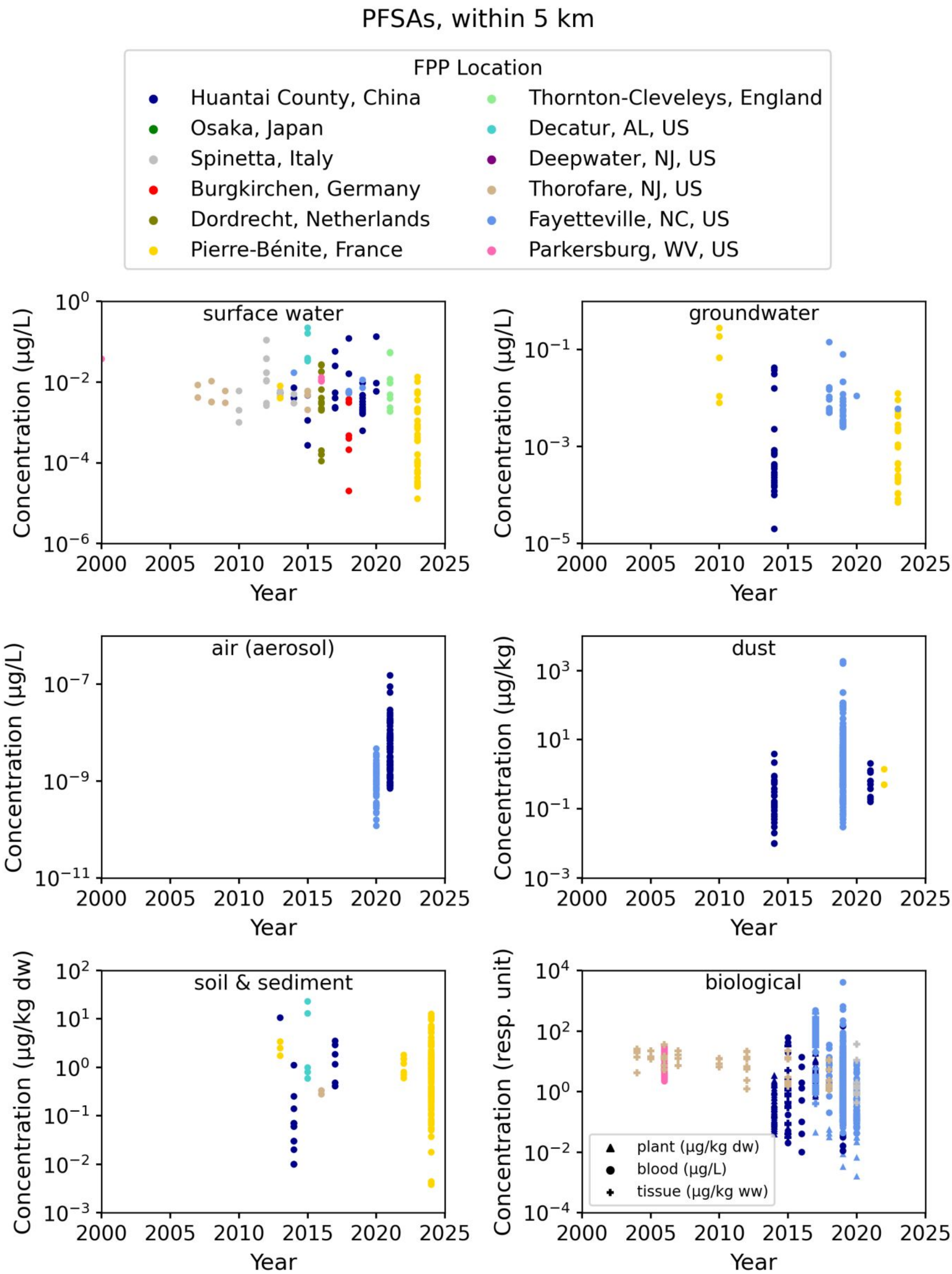

Figure S10. Corrected version of original Figure S11.

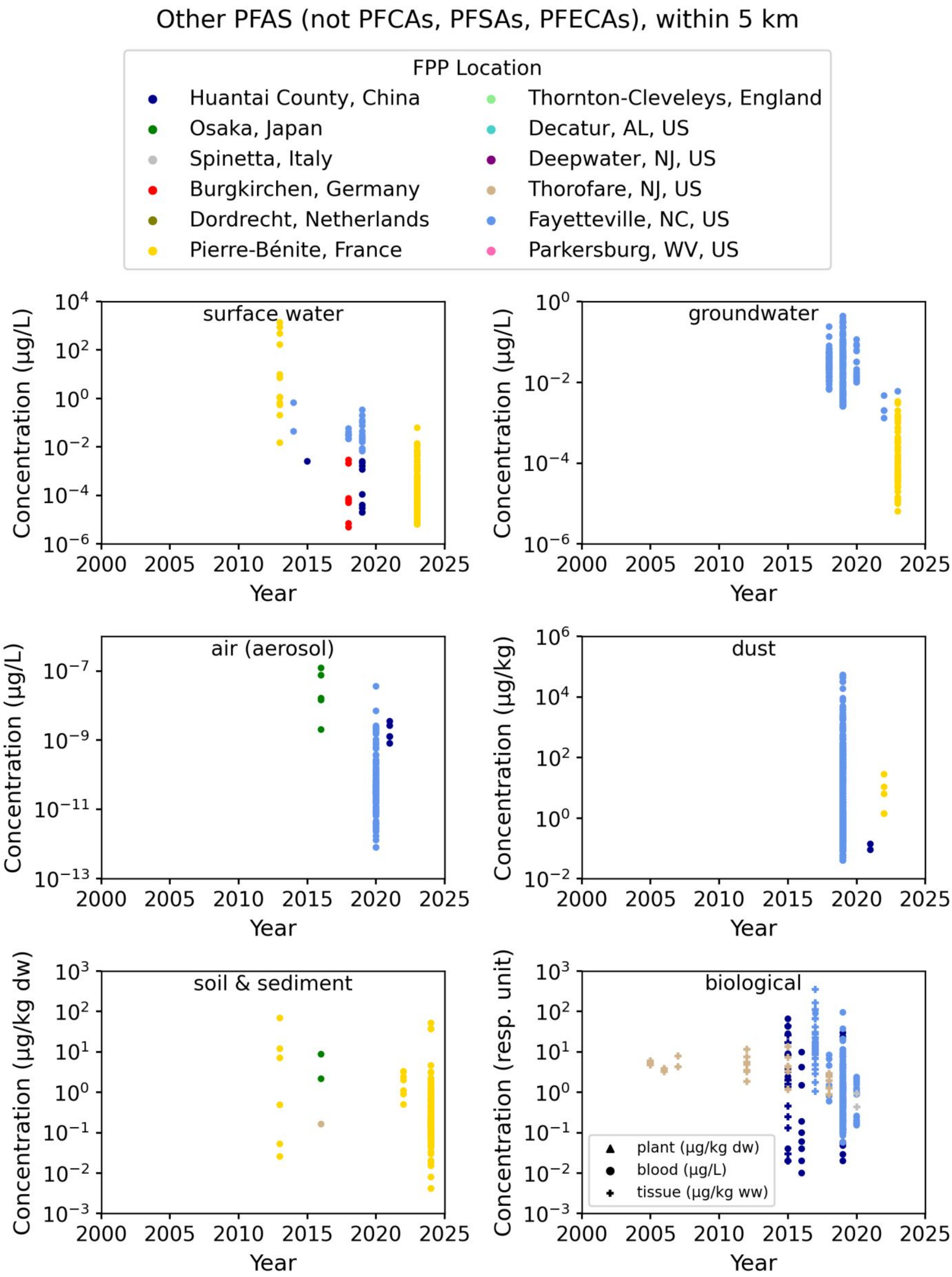

Figure S11. Corrected version of original Figure S12.

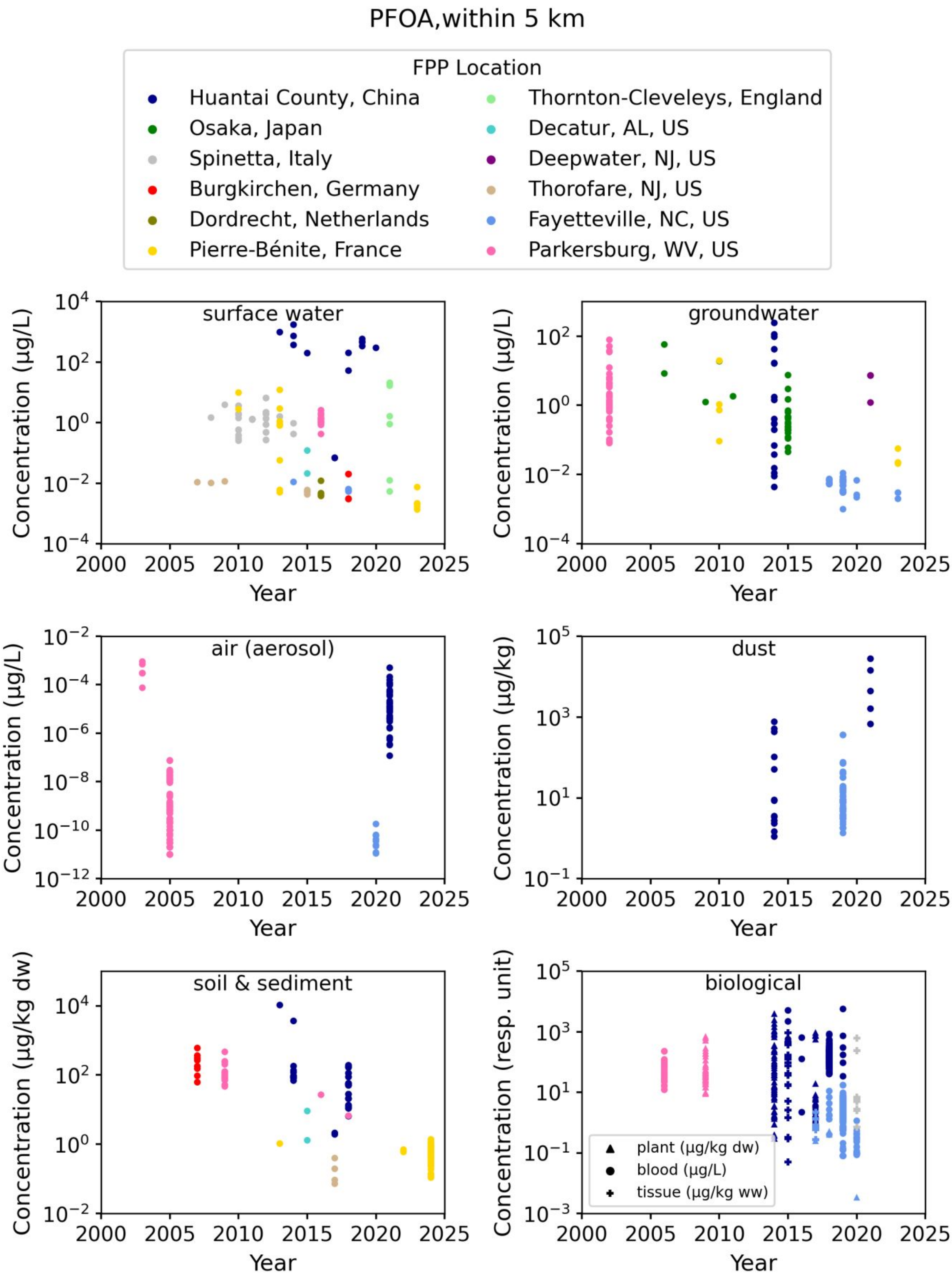

Figure S12. Corrected version of original Figure S13.

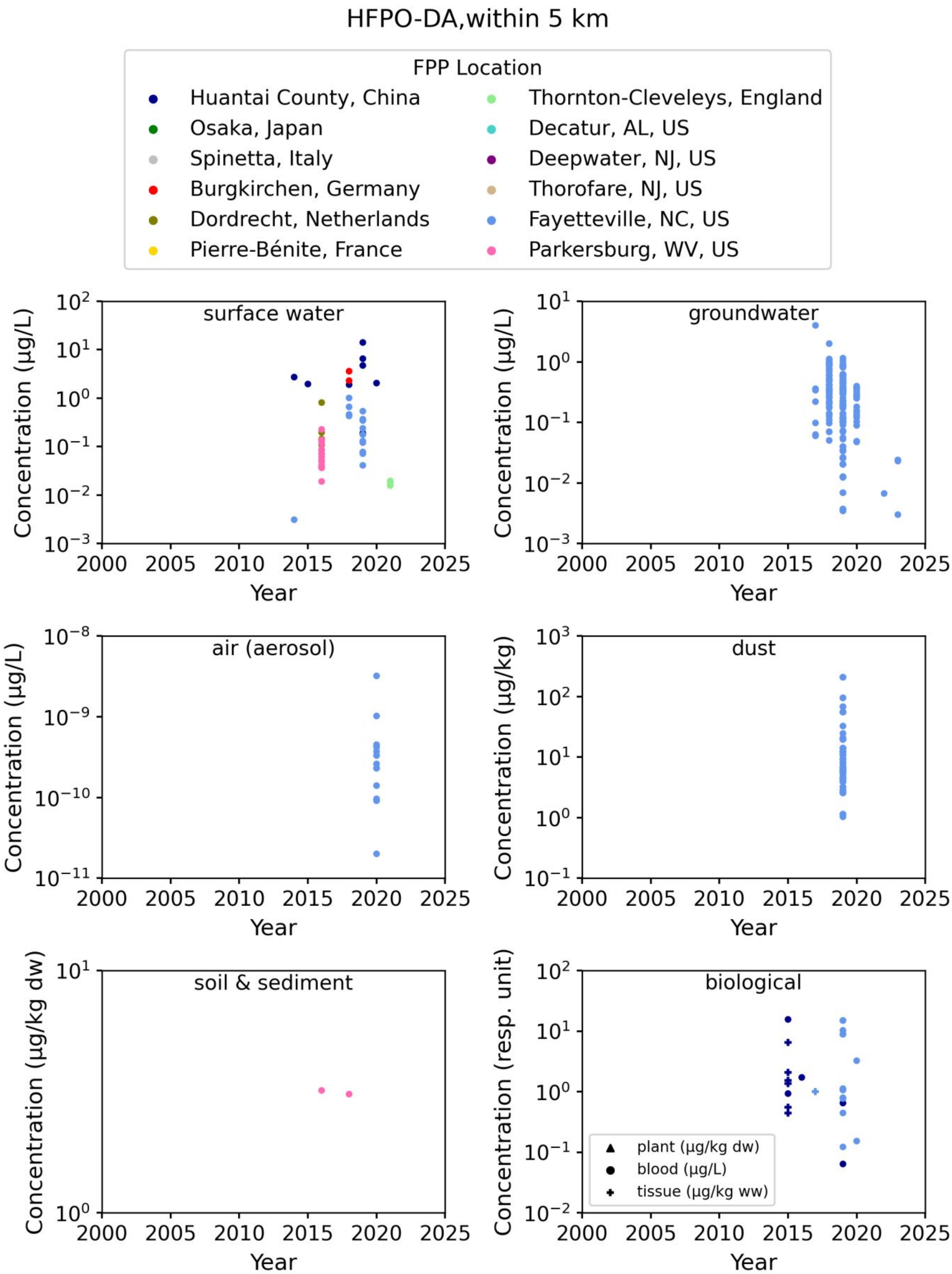

Figure S13. Corrected version of original Figure S14.

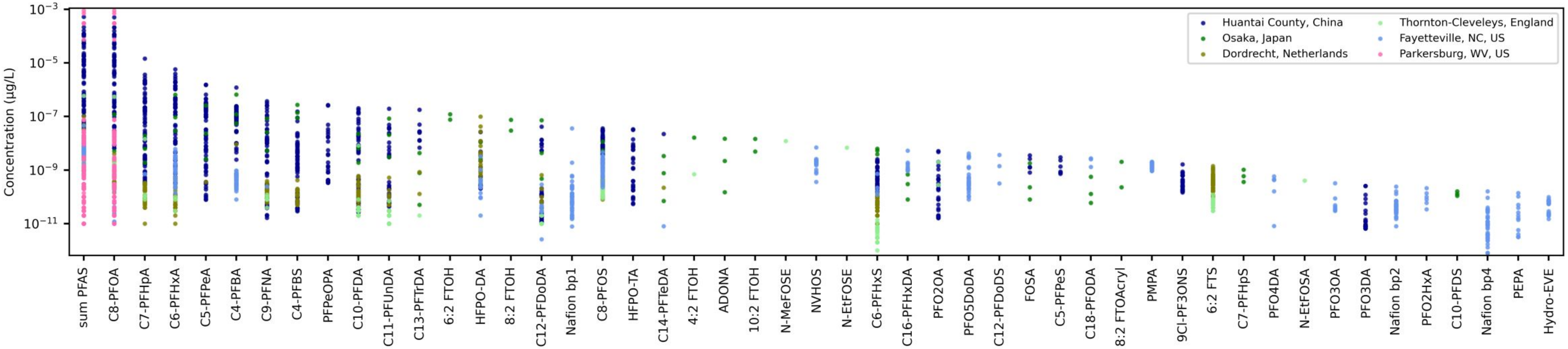

Figure S14. Corrected version of original Figure S17.
